# BAG3 Pro209 mutants associated with myopathy and neuropathy relocate chaperones of the CASA-complex to aggresomes

**DOI:** 10.1038/s41598-020-65664-z

**Published:** 2020-05-29

**Authors:** Elias Adriaenssens, Barbara Tedesco, Laura Mediani, Bob Asselbergh, Valeria Crippa, Francesco Antoniani, Serena Carra, Angelo Poletti, Vincent Timmerman

**Affiliations:** 1 Peripheral Neuropathy Research Group, Department of Biomedical Sciences, Institute Born Bunge, University of Antwerp, Antwerp, Belgium; 2 Dipartimento di Scienze Farmacologiche e Biomolecolari, Centro di Eccellenza sulle Malattie Neurodegenerative, Università degli Studi di Milano, Milano, Italy; 3 Department of Biomedical, Metabolic and Neural Sciences, University of Modena and Reggio Emilia, and Center for Neuroscience and Neurotechnology, Modena, Italy; 4 VIB-UAntwerp Center for Molecular Neurology, VIB and University of Antwerp, Antwerp, Belgium

**Keywords:** Biochemistry, Cell biology, Genetics, Diseases

## Abstract

Three missense mutations targeting the same proline 209 (Pro209) codon in the co-chaperone Bcl2-associated athanogene 3 (BAG3) have been reported to cause distal myopathy, dilated cardiomyopathy or Charcot-Marie-Tooth type 2 neuropathy. Yet, it is unclear whether distinct molecular mechanisms underlie the variable clinical spectrum of the rare patients carrying these three heterozygous Pro209 mutations in BAG3. Here, we studied all three variants and compared them to the BAG3_Glu455Lys mutant, which causes dilated cardiomyopathy. We found that all BAG3_Pro209 mutants have acquired a toxic gain-of-function, which causes these variants to accumulate in the form of insoluble HDAC6- and vimentin-positive aggresomes. The aggresomes formed by mutant BAG3 led to a relocation of other chaperones such as HSPB8 and Hsp70, which, together with BAG3, promote the so-called chaperone-assisted selective autophagy (CASA). As a consequence of their increased aggregation-proneness, mutant BAG3 trapped ubiquitinylated client proteins at the aggresome, preventing their efficient clearance. Combined, these data show that all BAG3_Pro209 mutants, irrespective of their different clinical phenotypes, are characterized by a gain-of-function that contributes to the gradual loss of protein homeostasis.

## Introduction

Protein homeostasis is maintained by a complex network of molecular chaperones and co-chaperones providing protection to client proteins at every stage of their life-time^1^. As soon as a nascent polypeptide leaves the ribosomal exit tunnel, chaperones interact with exposed domains to facilitate protein folding^2^. In case of protein misfolding, chaperones will either try to refold or guide the polypeptide towards degradation by proteasomes or the autophagy-lysosomal pathway^1^.

**Figure 1:**
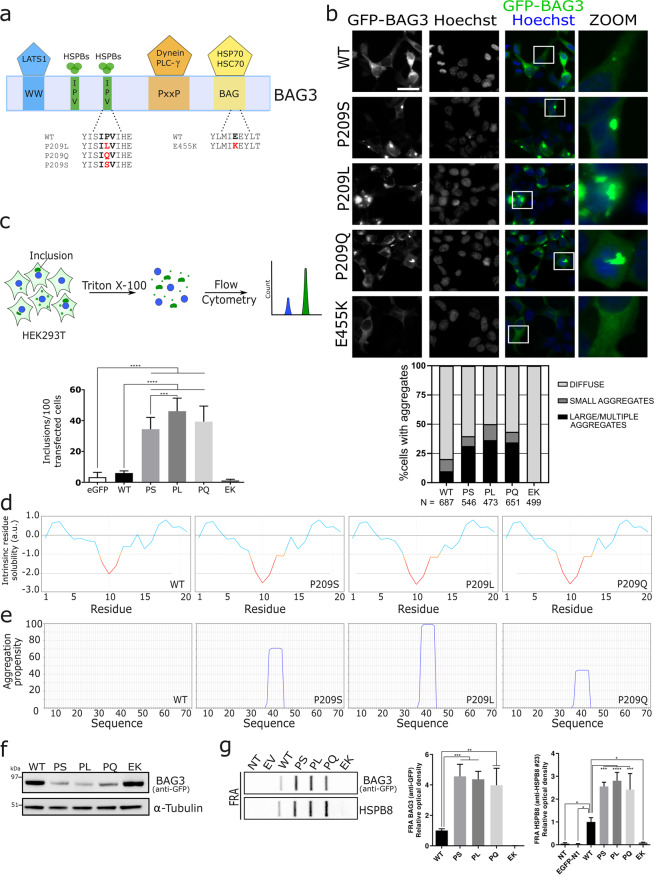
BAG3_Pro209 mutations cause cytoplasmic aggregation. (**a**) Schematic representation of the structure of BAG3, including the WW-domain, the two IPV-motifs, the PxxP-domain and BAG-domain. The known interactors of each motif are shown at the top and the missense mutations that were studied in this manuscript are shown at the bottom in red. (**b**) HEK293T cells stably expressing HSPB8-V5 were transiently transfected with BAG3-GFP constructs. Six random fields were selected for analysis. The mean number of cells counted per field was 95 and thus over 400 cells per genotype were counted. (scale bar = 10 μm) (**c**) Quantification of BAG3-GFP inclusions using Flow cytometric analysis of inclusions (FloIT). Transiently transfected HEK293T cells were collected and stained with DAPI prior to 0.1% Triton X-100 treatment. The intracellular BAG3-GFP inclusions and Hoechst-positive nuclei are subsequently quantified using flow cytometry. Bar graph represents the means of BAG3-GFP cytoplasmic inclusions per 100 transfected cells. One-Way ANOVA with Bonferroni’s multiple comparisons test were used for statistical analysis. (**d,e**) Bio-informatic analysis of (**d**) the solubility of wild type or mutant BAG3 with CamSol and (**e**) of the aggregation propensity with Tango software. (**f**) Western blot analysis of the NP-40 soluble fraction from HEK293T cells stably expressing HSPB8-V5 and transiently transfected with BAG3-GFP constructs. The constructs were abbreviated as followed: wild type (WT), Pro209Ser (PS), Pro209Leu (PL), Pro209Gln (PQ), Glu455Lys (EK). One of three representative western blots is shown. (**g**) Filter retardation assay (FRA) analysis of the NP-40 insoluble fraction. Anti-GFP and anti-HSPB8 antibodies were used to detect insoluble levels of BAG3 (wild type or mutants) and HSPB8. Relative optical densities are reported in the graphs as means ± SD of normalized values. One-Way ANOVA with Bonferroni’s multiple comparisons test were used for statistical analysis (n = 3). The constructs were abbreviated as followed: non-transfected (NT), empty vector (EV), wild type (WT), Pro209Ser (PS), Pro209Leu (PL), Pro209Gln (PQ), Glu455Lys (EK).

The activity of many chaperones is critically dependent on co-chaperones. One family of co-chaperones is represented by the Bcl2-associated athanogene (BAG) family of proteins, which in humans includes six members, encoded by 6 different genes^3^. All six family members share a conserved BAG-domain, which is essential for their binding to the Hsp70 chaperones^4^. BAG3 is a well-characterized family member that contains a number of additional protein domains besides the conserved BAG-domain, including two Ile-Pro-Val (IPV)-motifs, a PxxP domain and a WW-domain (Fig. 1a). Each of these domains is known to have specific interacting partners. For instance, the BAG-domain is known to mediate the interaction with Hsp70/Hsc70 or Bcl2^5–7^. The IPV-motifs have been shown to be indispensable for binding to small heat shock proteins (sHSPs)^8^, the WW-domain binds LATS1^9^, and the PxxP domain is necessary for the interaction with dynein and PLC-γ^10,11^.

The sHSP with the highest affinity for the IPV-motifs of BAG3 is HSPB8 (Hsp22)^12,13^. In fact, the protein stability of HSPB8 is critically dependent on BAG3, as it is rapidly degraded in its absence^14^. As also other members of the HSPB family are capable of binding to BAG3, it is thought that in case HSPB8 would be unable to fulfil its role (e.g. due to lower expression levels of HSPB8), these other sHSPs could partly replace its function by binding to BAG3. Such compensatory mechanisms would ensure that BAG3-sHSP interactions are maintained even under compromising conditions and underscore the importance of this interaction.

BAG3-HSPB8 is a sub-complex at the basis of a larger protein complex, known as the chaperone-assisted-selective-autophagy (CASA) complex. In addition to BAG3 and HSPB8, also Hsp70/Hsc70, CHIP and SQSTM1/p62 are part of this complex^15^. Under stress conditions, unfolded or misfolded proteins are rapidly recognized by sHSPs^16^, which are then transferred to ATP-dependent chaperones like Hsp70 for refolding. In case the substrate cannot be refolded by Hsp70, then the client is directed towards autophagosomal degradation by CHIP (an E3 ubiquitin ligase that ubiquitinylates the substrate^17–19^) and SQSTM1/p62 (an autophagy receptor that binds to ubiquitinylated proteins and transfers them to autophagosomes^20–22^). By clustering the different components into a single complex, substrates are likely handed over faster to reduce the potentially dangerous dwell time.

The complete substrate repertoire of the CASA-complex remains elusive. However, many model client proteins involved in the appearance of adult onset neurodegenerative diseases were shown to aggregate less in the presence of the CASA-complex, such as elongated polyQ-variants of the mutant androgen receptor (AR), mutant huntingtin (HTT) or mutant ataxin-3 (ATX-3), SOD1 mutants, TDP-43 mutants, neurotoxic dipeptides deriving from the expanded repeat of the C9ORF72 mRNA. At the very least this illustrates that the CASA chaperone complex can handle a diverse array of misfolded proteins^23^.

In light of its important role for the maintenance of cellular protein homeostasis, it is not surprising that mutations in the co-chaperone *BAG3* have been reported to cause a variety of disorders affecting distal muscles, cardiomyocytes or peripheral nerves. One hot-spot residue is the proline at codon 209 of BAG3. Genetic variants of this codon were previously linked to cardiomyopathy and distal myopathy^24,25^. More recently, also two families with late-onset axonal Charcot-Marie-Tooth (CMT) neuropathy were reported with a novel Pro209Ser mutation in *BAG3*^26^. The impact of the different Pro209 mutations on the function of BAG3 remains unclear. The Pro209Leu mutant, which causes early-onset dilated cardiomyopathy and/or severe distal myopathy, was shown to cause a toxic gain-of-function by impairing the Hsp70 client processing^27^. This results in the accumulation of aggregated proteins that sequester important protein quality control (PQC) factors, including Hsp70. Importantly, accumulation of aggregated proteins has been documented in the biopsies of patients affected by myopathy and peripheral neuropathy^24,28^, further supporting the interpretation that these mutations may affect BAG3 PQC functions.

Here, we studied the impact of the three heterozygous Pro209 missense mutations and compared it with the cardiomyopathy-causing BAG3_Glu455Lys mutant, which is located in the BAG-domain^29^, and the wild type BAG3 protein. We found that all BAG3_Pro209 mutants equally interact with the other CASA-components compared to wild type. However, due to their increased propensity to aggregate, all BAG3_Pro209 mutants acquire a toxic gain-of-function leading to the sequestration of all CASA-components, along with their bound poly-ubiquitinylated clients, in perinuclear aggregates called aggresomes. This, in turn, promotes a general collapse in the chaperone-network, as previously reported for the P209L mutant^27^.

## Results

We studied the pathogenic consequences of 3 previously reported BAG3 missense mutations, located in the second IPV-motif of BAG3 (Fig. 1a) which mediates the interaction with sHSP family members, and causing distinct phenotypes: Pro209Leu (early-onset dilated cardiomyopathy and/or severe distal myopathy^24^), Pro209Gln (late-onset distal myopathy^25^), Pro209Ser (late-onset CMT2^26^). In addition, we included the clinical and molecular well-characterised BAG3_Glu455Lys mutant^30^. The BAG3_Glu455Lys mutation is located in a different protein-domain of BAG3 (Fig. 1a), the BAG-domain which mediates the direct interaction with Hsp70, and causes dilated cardiomyopathy^29^.

### BAG3 Pro209-mutations cause BAG3 protein aggregation

To investigate the impact of the three IPV-mutations, we transiently overexpressed GFP-tagged wild type or mutant BAG3 in HEK293T cells that stably overexpress HSPB8. The expression of exogenous wild type BAG3 compared to endogenous BAG3 is about 5-fold higher after transient transfection of these HEK293T cells (Fig. S1). Of note, the HSPB8-BAG3-Hsp70 complex identified in HeLa cells has a stoichiometry corresponding to 2:1:1^16^. Thus, to maintain this stoichiometry, we stably overexpressed HSPB8 in HEK293T cells, which are characterized by low expression levels of HSPB8 and abundant Hsp70.

We performed fluorescence microscopy using these HEK293T cells that stably overexpress HSPB8 to verify the subcellular distribution of BAG3. The images showed a different localization and distribution of the three IPV-motif located BAG3_Pro209 mutants compared to wild type BAG3 and the BAG-domain located BAG3_Glu455Lys mutant (Fig. 1b). Both Glu455Lys and wild type BAG3 showed a predominantly diffuse cytoplasmic distribution (Fig. 1b). In contrast, BAG3_Pro209 mutants showed an aberrant distribution, with low levels of diffuse cytoplasmic BAG3 protein and higher levels of BAG3-GFP protein sequestered in multiple smaller aggregates or one irregular shaped large perinuclear aggregate. More specifically, 31.4% of cells transfected with Pro209Ser mutant, 36.5% of cells transfected with Pro209Leu and 34.4% of cells transfected with Pro209Gln mutant presented with large aggregates at 24 hours after transfection (Fig. 1b). From this quantification, subtle differences were detected between the three Pro209 mutants; as the Pro209Leu mutation caused aggregation in a slightly higher number of cells compared to the other mutants.

To confirm these results with an independent technique, we made use of a recently developed method to quantify cellular protein aggregates in a high-throughput manner, known as FloIT^31^. This method employs the fluorescence counting capabilities of a flow cytometer to determine the number of cellular aggregates in cellular lysates (Fig. 1c). HEK293T cells that stably overexpress HSPB8 were transiently transfected with the different BAG3-GFP proteins, lysed in a mild detergent (0.1% Triton X-100 in PBS) supplemented with DAPI for nuclear staining. GFP-positive aggregates were then investigated by FloIT (Fig. S2). All three IPV-mutants formed significantly more inclusions than wild type BAG3 or the BAG-domain Glu455Lys mutant (Fig. 1c). Similar to what we observed with fluorescence microscopy, the Pro209Leu mutant formed a higher amount of aggregates. Together, these data demonstrate that all three mutants affecting the IPV-motif cause protein aggregation, a phenotype that seems unique to IPV-mutants, as the BAG-domain Glu455Lys mutant and BAG3 wild type protein remained diffusely distributed in the cytoplasm.

To assess if this altered cytoplasmic distribution also affects the solubility of the protein, we first employed two bio-informatic prediction tools: CamSol^32^ and Tango^33^. Both methods predicted a strong reduction in protein solubility for each of the genetic mutants **(**Figs. 1d,e and S3), with the largest reduction in protein solubility for the Pro209Leu substitution. To determine whether these mutants are indeed less soluble, we extracted the proteins from cells overexpressing wild type or mutated BAG3 using a buffer that contains 0.5% of NP-40 as detergent (Fig. 1f). While the protein levels of the Glu455Lys mutant were very similar to those of wild type BAG3 in detergent soluble fractions, the levels of all three BAG3_Pro209 mutations (Ser, Leu and Gln) were drastically decreased in the NP-40 buffer. To confirm that these mutants become insoluble, we used a filter retardation assay (FRA). This showed that the Pro209 mutants were highly enriched in the NP-40 insoluble fraction (Fig. 1g). Interestingly, also HSPB8 was found in higher amounts in the insoluble fraction. Summation of both soluble and insoluble fractions shows that total levels of BAG3 are slightly increased for mutants (Fig. S4).

**Figure 2:**
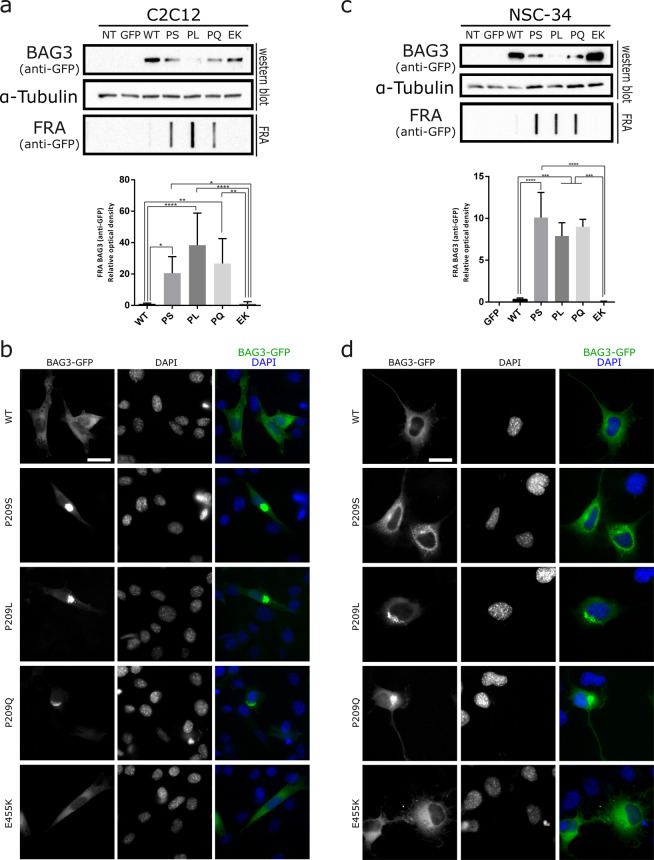
BAG3_Pro209 mutants also aggregate in muscle (C2C12) and motoneuron-like cells (NSC-34). We transiently transfected GFP-tagged BAG3 wild type or mutant constructs in C2C12 and NSC-34 cells. We then verified protein aggregation by separating the soluble fraction (western blot) and insoluble fraction (filter retardation assay (FRA)) (**a**,**c**) or verified protein aggregation by immunofluorescence (**b**,**d**). The FRA analysis is displayed for the NP-40 insoluble fraction. Relative optical densities are reported in the graphs as means ± SD of normalized values. One-Way ANOVA with Bonferroni’s multiple comparisons test were used for statistical analysis (n = 3). Scale bar = 10 µm.

Since the mutations affect the heart, muscle or peripheral motor neurons in patients, we verified whether the phenotypes described above are also observed in these cell types. To this end, we overexpressed GFP-tagged BAG3 in mouse myoblasts (C2C12 cells) and immortalized motor neurons (NSC-34 cells). The BAG3_Pro209 mutants also aggregated in C2C12 and NSC-34 cells, while BAG3 wild type or BAG3_Glu455Lys did not (Fig. 2).

Combined these data demonstrate that all BAG3_Pro209 mutants have a decreased protein solubility and this gives rise to large protein aggregates in the cytosol, regardless of the cell type investigated.

### The perinuclear aggregates formed by BAG3 Pro209 mutants are aggresomes

**Figure 3:**
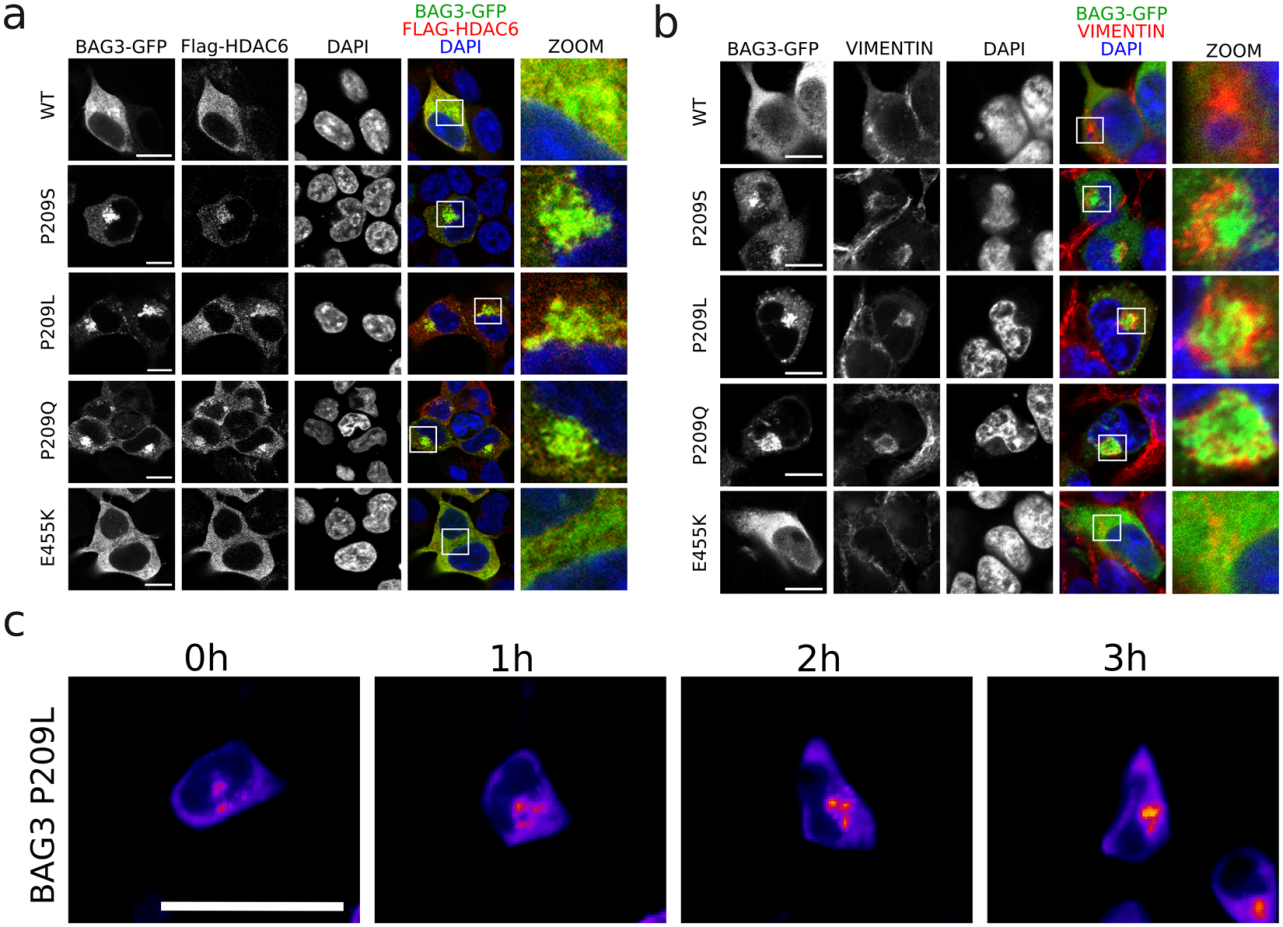
BAG3_Pro209 mutants accumulate at aggresomes. Co-localization was assessed between BAG3-GFP and aggresome-markers in HEK293T cells stably expressing HSPB8-V5 and transiently transfected for 24 h with BAG3-GFP constructs. As markers for aggresomes we used: (**a**) FLAG-HDAC6, and (**b**) endogenous vimentin. Scale bar = 10 µm. (**c**) Live-cell time-lapse imaging of GFP-tagged BAG3_Pro209Leu in HEK293T cells. Scale bar = 25 µm.

Since BAG3 aggregates have an irregular shape and BAG3 was previously shown to translocate to aggresomes^11^, we assessed whether these structures were aggresomes. As HDAC6 and vimentin are two well-known markers for aggresomes^34,35^, we verified whether the BAG3_Pro209 variants colocalized with HDAC6 or vimentin. Confocal images confirmed that the BAG3_Pro209 aggregates are positive for both HDAC6 (Fig. 3a) and vimentin (Fig. 3b), supporting the interpretation that BAG3_Pro209 mutants accumulate in the form of aggresomes.

To gain insight into the different stages of this process, we performed live-cell time-lapse imaging in HEK293T cells after transient transfection of GFP-tagged BAG3_Pro209Leu. We observed that mutant BAG3 first formed smaller aggregates at the periphery of the cell which clustered at one central spot near the nucleus over time (Fig. 3c). Similar results were obtained in HeLa cells, further suggesting that aggregation is an intrinsic property of this mutant (Fig. S5).

### BAG3 Pro209 mutants sequester chaperones of the CASA-complex at aggresomes

**Figure 4:**
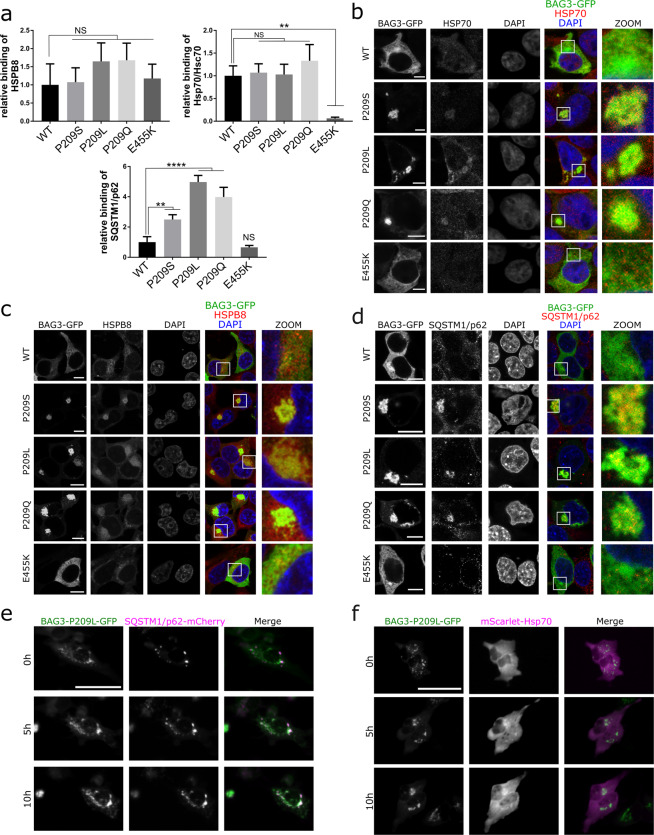
BAG3_Pro209 mutants sequester other members of the CASA-complex in aggresomes. HEK293T cells that stably overexpress HSPB8-V5 were transiently transfected with wild type or mutant BAG3-GFP constructs to assess the interaction between BAG3 and components of the CASA-complex. (**a**) Co-immunoprecipitation of BAG3-GFP and the CASA-complex using the GFP-trap system. The amount of interacting proteins was quantified and corrected for the amount of immunoprecipitated BAG3 as represented in the graph bar (means ± SD). One-Way ANOVA with Bonferroni’s multiple comparisons test were used for statistical analysis. NS = non-significant, **p < 0.01, and ****p < 0.0001 (n = 3). The wild type (WT) or mutants were abbreviated as followed: Pro209Ser (PS), Pro209Leu (PL), Pro209Gln (PQ), Glu455Lys (EK). (**b–d**) Immunocytochemistry of BAG3-GFP constructs to assess colocalization with (**b**) endogenous Hsp70, (**c**) HSPB8, and (**d**) SQSTM1/p62. Scale bar = 5 µm (**b**) and 10 µm (**c,d**). (**e,f**) Live-cell time-lapse imaging of GFP-tagged BAG3_Pro209Leu and RFP-tagged SQSTM1/p62 or Hsp70. HeLa cells were transiently transfected with mutant BAG3-GFP constructs and (**e**) mCherry-tagged SQSTM1/p62 or (**f**) mScarlet-tagged Hsp70. Cells were imaged once per hour. Scale bar = 50 µm.

BAG3 forms a stoichiometric complex with HSPB8 and Hsp70/Hsc70 and the Pro209 residue is located within the binding domain of BAG3 to HSPB8^8,14^. We therefore verified by co-immunoprecipitation whether the Pro209 mutations affect the ability of BAG3 to bind its binding partners HSPB8 and Hsp70/Hsc70 (Figs. 4a and S6). Our data show that none of the BAG3 mutants abolished the interaction with HSPB8, nor with Hsp70/Hsc70 (Figs. 4a and S6). The interaction with Hsp70/Hsc70 was only affected by the BAG3_Glu455Lys mutation, which is located within the BAG domain essential for binding Hsp70^30,36^. By performing the reverse experiment, assessing the co-immunoprecipitation of BAG3 along with HSPB8, we obtained similar results (Supplementary Fig. S7). By contrast, the interaction with the CASA-partner SQSTM1/p62 was increased by the Pro209 mutations compared to wild type BAG3 (Fig. 4a). This is consistent with a previous independent report^37^. So, although the IPV-motifs mediate the interaction with HSPB8, we found that Pro209 mutants primarily affect the interaction with SQSTM1/p62, which, as far as we know, is not mediated by a direct interaction between the two proteins, but requires the assembly of the full CASA-complex bound to poly-ubiquitin chain linked misfolded proteins.

To assess whether BAG3 relocates the CASA-complex to aggresomes, we performed co-localization experiments. Hsp70 and HSPB8 showed a diffuse cytoplasmic distribution in cells expressing BAG3 wild type or the Glu455Lys mutant (Fig. 4b,c). By contrast, in cells overexpressing BAG3_Pro209 mutants, we observed relocalization of both Hsp70 and HSPB8 to aggresomes (Fig. 4b,c), in line with the transition of HSPB8 from the NP-40 soluble to the insoluble fraction in a manner similar to BAG3 (Fig. 1g).

Next, we tested if mutant BAG3 aggresomes were also positive for sequestosome 1 (SQSTM1/p62). SQSTM1/p62 has the ability to bind both ubiquitin and protein degradation machinery and was found to regulate the formation of aggresomes^20–22,38^. Using confocal imaging, we observed that SQSTM1/p62 indeed colocalizes with the perinuclear BAG3 aggregates formed by all three Pro209 mutants, while SQSTM1/p62 maintained its typical disperse pattern in cells overexpressing wild type or Glu455Lys mutant BAG3 (Fig. 4d). Other members of the CASA-complex thus relocate to the aggresome in cells expressing BAG3_Pro209 mutants.

To investigate when Hsp70 and SQSTM1/p62 are recruited to the BAG3-aggregates, we performed live-cell time-lapse imaging of transiently transfected HeLa cells. We co-transfected GFP-tagged BAG3 with mCherry-tagged SQSTM1/p62 or mScarlet-tagged Hsp70. We found that both Hsp70 and SQSTM1/p62 co-localized with BAG3 in pre-aggresome bodies, which were transported over time towards the maturing aggresome (Fig. 4e,f). This supports the interpretation that Hsp70 and SQSTM1/p62 associate with BAG3 already in the early stages of the aggregation process. Note that we could not verify HSPB8, as tagging the small protein with a fluorescent protein of the same size, could potentially interfere with its functioning.

In summary, as a consequence of its increased aggregation propensity, mutant BAG3_Pro209 relocates Hsp70, HSPB8 and SQSTM1/p62 to the aggresomes, potentially decreasing their availability and compromising their functioning.

### BAG3 Pro209 mutants are trapped at aggresomes due to slower subunit exchange between the soluble and insoluble fraction

**Figure 5:**
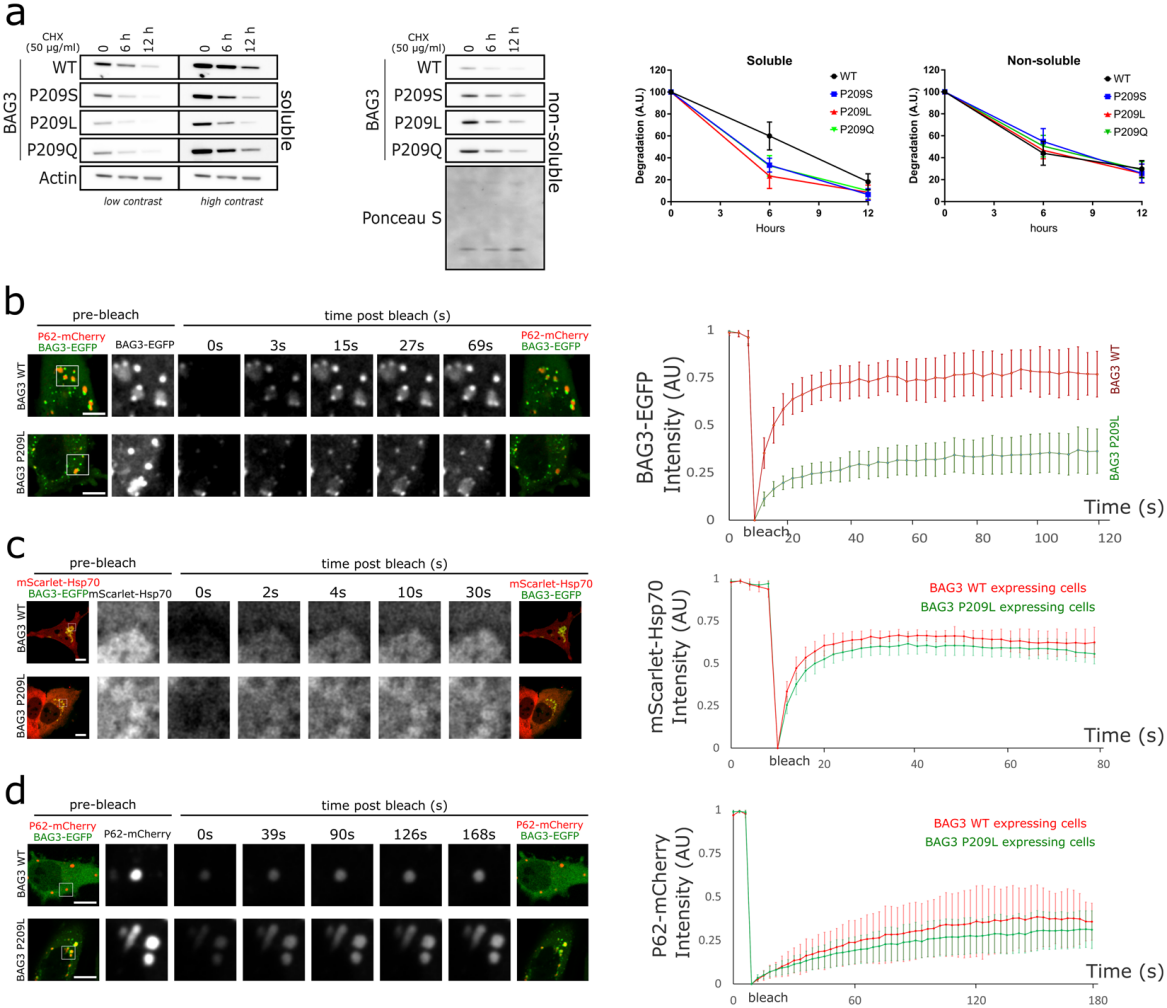
BAG3_Pro209 mutants are trapped in long-lasting aggresome structures due to reduced subunit exchange. (**a**) Protein degradation rates were determined with a cycloheximide wash-out experiment. HEK293T cells that stably overexpress HSPB8-V5 were transiently transfected with wild type or mutant BAG3-GFP constructs and subjected to cycloheximide treatment (50 µg/ml) for the indicated time. Protein turnover of BAG3-GFP was determined by western blot after separation of the soluble from insoluble fraction. (n = 3). (**b–d**) Fluorescence recovery after photobleaching (FRAP) analysis was performed on HeLa cells that were transiently transfected with BAG3-GFP and mScarlet-Hsp70 or SQSTM1/p62-mCherry constructs. Bleaching was performed either on (**b**) BAG3-GFP, (**c**) mScarlet-Hsp70, or (**d**) SQSTM1/p62-mCherry. Quantification of the fluorescence intensity over time was plotted for cells overexpressing WT and mutant BAG3. Graph bar shows the means (±SD) over time (n = 6). Scale bar = 10 µm.

The increased aggregation propensity of the BAG3_Pro209 mutants and their accumulation at the aggresome, may compromise their turnover. To this end we first verified whether mutant BAG3 was still degraded by autophagy^15^ and found that this is indeed the case (Fig. S8). Next, we performed a cycloheximide wash-out experiment, which allowed us to determine the degradation rate of BAG3. At time point zero, 6 and 12 hours after cycloheximide treatment, we collected protein lysates and separated the soluble from insoluble fractions to be able to specifically study the protein turnover of the non-soluble aggresome-enriched fraction. We found that BAG3_Pro209 mutants were enriched in the non-soluble fraction (Fig. 5a), in line with Fig. 1. However, while wild type BAG3 had a comparable depletion curve in the soluble and insoluble fraction, mutant BAG3 was depleted faster in the soluble fraction compared to the insoluble fraction (Figs. 5a and S9). This suggests that mutant BAG3 is either degraded faster or undergoes an increased transitioning from the soluble to the insoluble fraction. Given our other results which demonstrate that mutant BAG3 accumulates in the insoluble fraction, the latter seems the most plausible explanation.

To gain further insight in the dynamics of BAG3 at aggresomes, we performed fluorescence recovery after photo-bleaching (FRAP) experiments on HeLa cells overexpressing wild type or mutant BAG3-GFP. This allowed us to assess whether aggresomes still exchange BAG3-subunits with the pool of soluble cytosolic proteins, providing information on the solubility of these inclusions. Note that in cells overexpressing wild type BAG3 the number of cells with aggresome-like structures is very low, as we showed in Fig. 1. However, for the FRAP experiments, we specifically selected this minority of cells in order to be able to compare the recovery rates in BAG3-positive inclusions. The FRAP-measurements demonstrated that wild type BAG3 recovered rapidly after photobleaching of small cytoplasmic inclusions, demonstrating its dynamic behaviour and rapid exchange between compartments (Fig. 5b). For mutant BAG3, we observed that a large proportion of aggresome-associated BAG3_Pro209Leu mutant is immobile. Furthermore, compared to wild type BAG3, the exchange rate of the mobile BAG3_Pro209Leu mutant subunits is drastically slower (Fig. 5b). We noted that a number of cells were still forming aggresomes with pre-aggresome bodies spread across the cytoplasm, of which there appeared to be two types: one type is positive for SQSTM1/p62 and the other type is negative for SQSTM1/p62. We therefore repeated the FRAP experiment and compared the recovery rate of BAG3_Pro209Leu in SQSTM1/p62-positive pre-aggresomes versus SQSTM1/p62-negative pre-aggresomes. The fluorescence recovery of BAG3 was not different between SQSTM1/p62-positive and SQSTM1/p62-negative pre-aggresome bodies (Fig. S10), indicating that the presence of SQSTM/p62 is not influencing BAG3 mobility.

As our data show that mutant BAG3 is trapped in aggresomes and that our co-localization data show that other members of the CASA-complex are also present in these aggresomes, we verified whether mutant BAG3 also disturbed the subunit exchange rate of other members of the CASA-complex. We therefore performed FRAP on aggresome-like structures in cells overexpressing wild type or mutant BAG3 and found that neither Hsp70 nor SQSTM1/p62 had altered fluorescence recovery rates, which suggests that their subunit exchange and mobility is not altered by mutant BAG3 (Fig. 5c,d).

Together, these data show that two distinct pools of mutant BAG3 exist: one pool of mutant BAG3 is trapped in aggresome-associated structures with drastically reduced subunit exchange compared to wild type BAG3, while a second pool of mutant BAG3_Pro209Leu is moving freely within the cytosol. Due to a reduced exchange with the cytosolic (soluble) fraction, initial engagement with pre-aggresome bodies commits mutant BAG3 towards the aggresome, where it holds a residence time in the range of hours. This process occurs independently of SQSTM1/p62 recruitment at the BAG3 pre-aggresome bodies.

### BAG3 Pro209 mutants reduce the chaperone-capacity of the CASA-complex

Meister-Broekema et al. (2019) showed that BAG3_Pro209 mutants fail to stimulate Hsp70-dependent client processing, leading to the sequestration of ubiquitinylated Hsp70-bound clients into aggregates. We verified whether the aggresomes formed by all BAG3_Pro209 mutants were enriched for ubiquitinylated proteins, which would suggest a failure to degrade Hsp70-bound clients.

**Figure 6:**
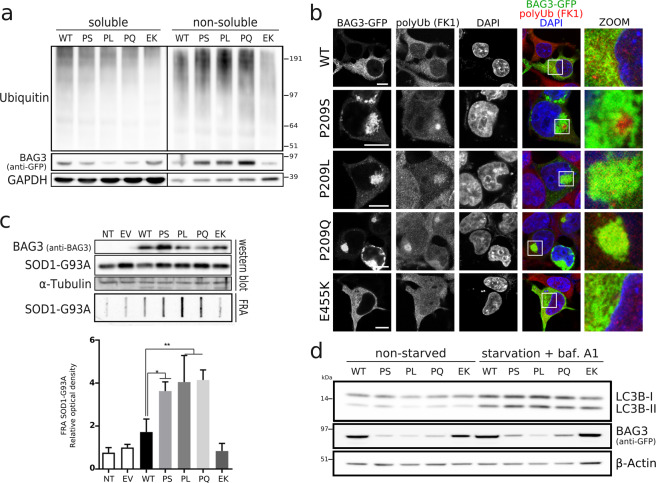
BAG3_Pro209 mutations cause a failure in chaperone-function of the CASA-complex. Chaperone-activity was assessed in HEK293T cells stably expressing HSPB8-V5 and transiently transfected with wild type or mutant BAG3-GFP constructs. (**a**) Aggregation of ubiquitinylated clients was verified by separation of the soluble and insoluble fraction. Both fractions were analyzed by western blot with anti-ubiquitin as marker for the accumulation of ubiquitinylated-proteins. (**b**) Immunocytochemistry of BAG3-GFP constructs to assess colocalization with ubiquitinylated proteins. Scale bar = 10 µm (**c**) Protein aggregation assay by transient transfection of model client protein SOD1_G93A. The same total protein lysates were analyzed by western blot and filter retardation assay (FRA). Relative optical densities are reported in the graph as means ± SD of normalized values. One-Way ANOVA with Bonferroni’s multiple comparisons test were used for statistical analysis. *p < 0.05, **p < 0.01 (n = 3). (**d**) Autophagic activity was determined by western blot, before and after starvation by serum depletion plus 10 nM bafilomycin A1 for 2 hours. Protein lysates were analyzed by SDS-PAGE with LC3B-II as a marker for autophagosomes. Following abbreviations were used: non-transfected (NT), empty vector (EV), wild type (WT), and BAG3 mutations Pro209Ser (PS), Pro209Leu (PL), Pro209Gln (PQ), Glu455Lys (EK).

To this end, we transiently transfected HEK293T cells that stably overexpress HSPB8-V5 and separated the soluble from the insoluble fraction. We found that the insoluble fraction from cells expressing BAG3_Pro209 mutants contained more ubiquitinylated proteins (Fig. 6a). Using confocal microscopy, we confirmed that these insoluble ubiquitinylated proteins cluster at the aggresome (Fig. 6b). Interestingly, the amount of ubiquitinylated proteins in the soluble fraction was the same as for wild type BAG3 (Fig. 6a), suggesting that clients are still recognized by the CASA-complex, but that a failure in client processing leads to accumulation of ubiquitinylated proteins at aggresomes.

To further test the hypothesis that the BAG3_Pro209 mutants acquire a toxic gain of function that ultimately impairs the clearance of aggregation-prone proteins, we studied the degradation of a well-characterized model client known to be targeted for autophagy-mediated clearance by the CASA-complex^39^. To this end, we co-transfected SOD1_G93A together with wild type or mutant BAG3. While the soluble levels of SOD1_G93A were similar in cells expressing the different BAG3 variants, we detected a significantly higher amount of SOD1_G93A in the insoluble fraction in cells expressing the three Pro209 mutants (Fig. 6c), further suggesting that the misfolded proteins are still recognized by the CASA-complex but fail to be degraded. We next tested another known CASA-complex substrate, the peptide poly-GA, an aggregation-prone dipeptide repeat protein produced from the ALS-linked *C9orf72* gene^40^. Similar to SOD1_G93A, the degradation of poly-GA was impaired in cells overexpressing BAG3_Pro209 mutants (Fig. S11).

So far our data argue against the possibility that failure to degrade their clients by BAG_Pro209 mutants is due to the inability of the CASA-complex to recognize the clients, suggesting that the client is recognized and bound by the CASA-complex containing BAG3_Pro209 mutants, but that clients are no longer released for degradation by the autophagosomes. Alternatively, the BAG3_Pro209 mutants impair the autophagy degradation pathway, which would also lead to an accumulation of misfolded client proteins as the aggresome is highly enriched in autophagosomal structures and this route is used for client degradation. To distinguish between these two possibilities, we verified whether the autophagic flux was impaired. As shown in Fig. 6d, the autophagic pathway is not impaired by BAG3_Pro209 mutants, suggesting that the accumulation of ubiquitinylated proteins cannot be explained by impairment of autophagy and supporting the idea that the CASA-complexes composed of BAG3_Pro209 mutants fail to release the bound client from Hsp70 for degradation by autophagosomes. This interpretation is in line with Meister-Broekema *et al*. (2019), who showed that BAG3_Pro209Leu fails to stimulate Hsp70-dependent client processing.

### HDAC6 interference does not prevent aggresome formation by BAG3 Pro209 mutants

Since the BAG3_Pro209 mutations lead to accumulation of ubiquitinylated clients at aggresomes due to a failure in client degradation, we verified whether interference with the aggresome-formation pathway could be pursued as a therapeutic strategy. To this end, we focused on the histone deacetylase HDAC6 for two reasons: (i) it was previously shown that HDAC6 is essential for aggresome formation upon proteasome inhibition^35^, and (ii) HDAC6-inhibitors have shown promising results as a therapeutic strategy in the field of motoneuron and neuromuscular disorders^41–45^.

**Figure 7:**
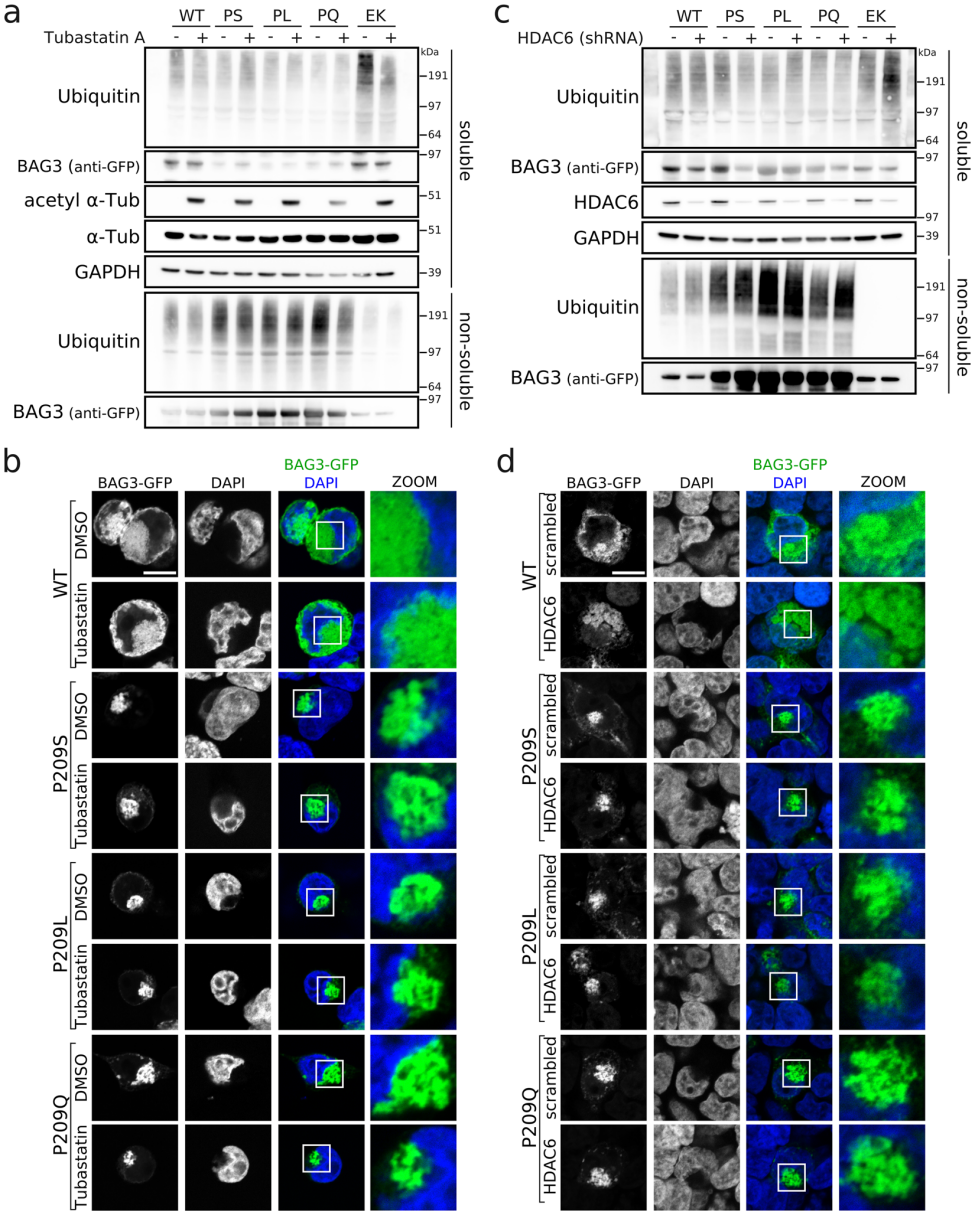
HDAC6-inhibition with tubastatin A or HDAC6-depletion with shRNA does not rescue BAG3_Pro209-associated phenotypes. The protein aggregation and aggresome formation of BAG3_Pro209 mutants was assessed in HEK293T cells stably expressing HSPB8-V5 and transiently transfected with wild type or mutant BAG3-GFP constructs before and after HDAC6 inhibition (**a,b**), or depletion by shRNAs (**c,d**). Following abbreviations were used: wild type (WT), and BAG3 mutations Pro209Ser (PS), Pro209Leu (PL), Pro209Gln (PQ), Glu455Lys (EK). Scale bar = 10 µm.

We inhibited HDAC6 with Tubastatin A, which is an inhibitor that binds HDAC6 specifically but has no activity towards other HDACs^41^, and we verified the aggresome formation and protein aggregation in HEK293T cells. To ensure HDAC6 was inhibited prior to the aggresome formation by mutant BAG3, we started the Tubastatin A treatment two hours before transfection of wild type or mutant BAG3 plasmids. The effectiveness of the treatment was confirmed by the increase in tubulin acetylation, as HDAC6 is well known to deacetylate tubulin^46,47^. However, HDAC6 inhibition with Tubastatin A did not prevent the aggregation of mutant BAG3, the accumulation of ubiquitinylated proteins in the insoluble fraction, or the formation of aggresomes (Fig. 7a,b).

Although Tubastatin A effectively inhibited the deacetylase function of HDAC6, we wanted to rule out that other protein domains of HDAC6 were still contributing to aggresome formation. To this end we generated a stable knockdown line for HDAC6 by lentivirally transducing a short hairpin RNA in HEK293T cells stably expressing HSPB8-V5. We expressed wild type or mutant BAG3 in this HDAC6-knockdown line and, despite the drastic reduction in HDAC6 protein levels, the protein aggregation of mutant BAG3, the accumulation of ubiquitinylated client proteins in the insoluble fraction, and the formation of aggresomes were not prevented by depletion of HDAC6 (Fig. 7c,d).

Therefore, neither pharmacological inhibition nor genetic depletion of HDAC6 prevented aggresome formation in BAG3_Pro209 mutant cells. Inhibition of HDAC6 may therefore not offer the desired therapeutic potential to rescue the compromised chaperone-function in cells expressing BAG3_Pro209 mutants. Moreover, these data suggest that BAG3_Pro209 mutants induce aggresome formation downstream of HDAC6 or from an independent pathway.

## Discussion

Aggresome formation is a cellular response to an overload of misfolded proteins^34^. It involves many components from PQC factors, such as SQSTM1/p62 and chaperones, to cytoskeletal elements such as γ-tubulin and vimentin. The latter seem required for the clustering of the misfolded proteins^34^. This effort to group misfolded proteins at one well-determined spot ensures that potentially toxic proteins are removed from the remaining cytosol and protects the cell from adverse effects. The aggresome is therefore rich in ubiquitinylated proteins and requires chaperones and autophagosomes to remove and degrade these components in a controlled manner.

**Figure 8:**
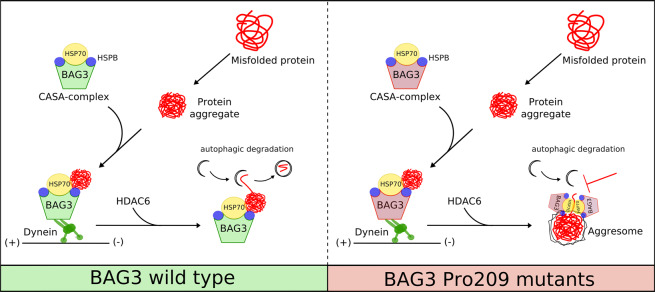
Schematic summary. Misfolded proteins are captured by the CASA-complex and transported to the MTOC, where autophagosomes are concentrated and efficiently degrade the misfolded cargo. BAG3_Pro209 mutations destabilize the protein’s intrinsic stability and lead to BAG3 aggregation. Moreover, due to a toxic gain-of-function, Pro209Leu BAG3 impairs the functional chaperone-cycle of Hsp70 (Meister-Broekema *et al.*, 2018). As a consequence, the CASA-complexes that contain mutant BAG3 accumulate at the aggresome with their bound clients and co-factors, preventing on the one hand the degradation of the Hsp70-bound misfolded cargo and sequestering important proteostasis factors such as HSPB8, SQSTM1/p62 and ubiquitin.

BAG3 is a scaffolding constituent that clusters different components of the PQC system into one protein complex. Upon inhibition of proteasomes, the BAG3-complex becomes activated and translocates to aggresomes to deliver ubiquitinylated proteins for degradation^11^. In this study, we found that disease-associated BAG3-mutations of Pro209 decrease the protein solubility leading to the aggregation of BAG3 and associated factors (Fig. 8). As a consequence, this leads to the formation of aggresomes, which are not only rich in BAG3 but also Hsp70, HSPB8 and ubiquitinylated substrates. We found that a reduction in exchange of BAG3 between soluble and insoluble pre-aggresomal puncta underlies the clustering of BAG3 at the aggresome. Due to this slower exchange, the initial engagement of BAG3 with non-soluble compartments commits BAG3 towards the forming aggresome. As such, BAG3 but also Hsp70, HSPB8 and the ubiquitinylated substrates that are bound by Hsp70 and HSPB8 are all transported towards the aggresome. This leads to clustering of ubiquitinylated species at the aggresome where a failure in the Hsp70-cycle, due to mutations in BAG3 as shown by Meister-Broekema *et al*. (2019), prevents the ubiquitinylated proteins from being degraded. This failure has been suggested to have important implications for cell function and disease. For example, the CASA complex facilitates the removal of filamin, which is essential for muscle maintenance^15^. The BAG3_Pro209Leu mutant is unable to properly clear damaged filamin; this, in turn, leads to its accumulation in form of aggregates, which might contribute to muscle cell dysfunction in BAG3_Pro209Leu patients^15^.

The chaperone-failure of the Hsp70 processing cycle is a surprising finding given that the mutations reside in a highly conserved motif for sHSP binding, while not affecting the HSPB8-BAG3 association^27^. Of note, binding of BAG3 to Hsp70 is not affected either by the Pro209 mutations (this study and^27^). This raises two important questions: (i) is the processing of HSPB8-specific clients also affected by the BAG3_Pro209 mutations? The fact that mutations in the *HSPB8* gene are linked to muscle atrophy^48^, together with the finding that the function and stability of HSPB8 depend on BAG3^14^, may suggest that altered Hsp70-BAG3 mediated processing of HSPB8-specific clients may have an impact on skeletal muscle function. (ii) To which extent do the IPV-motifs contribute to the chaperone-function of the CASA-complex? One way to test this would be by developing a mouse model that has the two IPV-motifs in BAG3 deleted, similarly to what has been developed for *in vitro* experiments^8^. This may then provide new insights in the diverse compositions and functions of the CASA-complex and help in understanding why IPV-mutations give rise to such diverse clinical phenotypes.

A limitation in studying the CASA-complex is that the substrate repertoire has not yet been fully elucidated. Assessing the activity of the CASA-complex is therefore limited to model substrates, which are often mutant proteins that misfold and aggregate. A concern to such approaches is that the overexpression of mutant BAG3 and mutant model substrates may by themselves overwhelm the degradation systems, while the PQC systems in patients with BAG3 mutations are typically not challenged by an additional mutant protein (such as SOD1_G93A or poly-GA). It will therefore be an important step in the future to assess whether the decrease in the activity of the CASA-complex, as reported in this study, can be translated to the affected tissues *in vivo*.

Given the distinct clinical phenotypes associated with the different BAG3_Pro209 mutants, the similarity in response of the different Pro209 mutants in our cellular and biochemical assays is noteworthy. The only difference we observed was that the Pro209Leu has a mildly increased propensity to aggregate compared to the two other Pro209 mutants and the Pro209Leu mutant was also the one affecting the clearance of SOD1_G93A the most. Note that the Pro209Leu mutation is also associated to the most severe phenotype with a very early onset^24^. However, it does not fully explain why this variant is associated with cardial symptoms, while the two other variants are more frequently linked to distal myopathy or peripheral neuropathy. In fact, there is even one patient reported with a Pro209Leu variant who only suffers from myofibrillar myopathy but not cardiomyopathy^49^, and the mouse does not seem to recapitulate this cardial phenotype either as a transgenic knock-in model of the BAG3_Pro215Leu mutation, equivalent to the human Pro209Leu mutation, did not show abnormal cardial function or morphology up to 16 months of age^50^. Similar to SQSTM1/p62 mutations, to which BAG3_Pro209 mutants bind stronger, genotype-phenotype correlations thus only poorly predict the clinical presentation^51^. However, the possibility that other modifying or (epi-) genetic factors contribute to clinical differences in both BAG3 and SQSTM1/p62 linked diseases cannot be excluded.

To conclude, despite the distinct phenotypes associated with Pro209 mutations in BAG3, they all seem to induce aggresome formation causing the sequestration of PQC factors. This suggests that, if a therapy for one of the Pro209-associated diseases can be identified, it may also be beneficial to other Pro209-associated phenotypes.

## Materials and Methods

### In vitro mutagenesis

Mutations were introduced through site-directed mutagenesis using the wild type BAG3-GSGS-GFP construct in the pEGFP-N1 vector (a kind gift of Josée N. Lavoie). Point mutations were introduced with following primers:

(Pro209Ser):

Fw: CGCGGGGGTACATCTCCATTTCGGTGATACACGAGCAGAA

Rv: TTCTGCTCGTGTATCACCGAAATGGAGATGTACCCCCGCG

(Pro209Leu):

Fw: CGCGGGGGTACATCTCCATTCTGGTGATACACGAGCAGAA

Rv: TTCTGCTCGTGTATCACCAGAATGGAGATGTACCCCCGCG

(Pro209Gln):

Fw: CGCGGGGGTACATCTCCATTCAGGTGATACACGAGCAGAA

Rv: TTCTGCTCGTGTATCACCTGAATGGAGATGTACCCCCGCG

(Glu455Lys):

Fw: AAAAAGTACCTGATGATCAAAGAGTATTTGACCAAAGAGC

Rv: GCTCTTTGGTCAAATACTCTTTGATCATCAGGTACTTTTT

Incorporation of the respective mutations was verified by Sanger sequencing.

### Generation of stable cell lines

HEK293T cells were transduced with lentivirus containing the HSPB8 ORF (NM_014365) in pLENTI6/V5 (Life Technologies, Carlsbad, CA, USA). In brief, HEK293T cells (purchased from ATCC, Teddingtin, Middlesex, UK) were transiently transfected with packaging (pCMV dR8.91), envelope (pMD2-VSV) and pLenti6/V5 plasmids using linear polyethylenimine (PEI) (23966-1, PolySciences Europe, Hirschberg an der Bergstrasse, Germany) or PEI MAX (24765-1, PolySciences Europe, Hirschberg an der Bergstrasse, Germany). After 48 h, the virus containing supernatant was collected, filtered and transferred to fresh HEK293T cells for infection. Positive cells were selected by blasticidine selection. Cells were cultured at 37 °C and 5% CO_2_ in DMEM (Life Technologies, Carlsbad, CA, USA) supplemented with 10% Fetal Bovine Serum, 1% Glutamine and 1% Penicillin-Streptomycin (Life Technologies, Carlsbad, CA, USA).

### Western blot and Filter retardation assay

For BAG3 solubility assessment in western blot (WB) and filter retardation assays (FRA) (Fig. 1), stable transfected HEK293T with HSPB8-V5 were plated at 90,000 cells/well, C2C12 at 65,000 cells/well, NSC-34 at 90,000 cell/well in 12-well plates. After 24 h, stable transfected HEK293T with HSPB8-V5 were transiently transfected using Lipofectamine3000/P3000 reagent with pEGFP-N1 as mock or BAG3-GFP constructs (wild type or mutants) alone or co-transfected with plasmid encoding SOD1_G93A (kindly provided by Dr. Caterina Bendotti, Mario Negri Institute, Milan, Italy) or poly-GA (kindly provided by Prof. Daisuke Ito, Keio University School of Medicine, Tokyo, Japan), following the manufacturers’ instructions (L3000-001; Life Technologies, Carlsbad, CA, USA). C2C12 cells were transiently transfected using Lipofectamine3000/P3000 reagent, NSC-34 were transiently transfected using Lipofectamine (18324010; Life Technologies, Carlsbad, CA, USA) transferrin (Sigma-Aldrich) with pEGFP-N1 as mock or BAG3-GFP constructs (wild type or mutants). Samples were harvested and centrifuged 5 min at 100 *g* at 4 °C. Cells were resuspended in NP-40 lysis buffer (150 mM NaCl, 20 mM TrisBase, NP-40 0.05%, 1.5 mM MgCl_2_, Glycerol 3%, pH 7.4) added DTT and Complete Protease inhibitor (Roche Applied Science, Indianapolis, IN, USA), and passed through a syringe 10 times. Lysed cells were centrifuged at 16,100 *g* for 15 min. Supernatants were collected and pellets resuspended in the same volume of NP-40 buffer without protease inhibitors and DTT, and finally sonicated. For the evaluation of the effects of BAG3 mutations on its chaperone-activity towards aggregation-prone proteins (SOD1_G93A) (Fig. 6), HEK293T cells were co-transfected with BAG3-GFP constructs and SOD1_G93A encoding plasmid, as described above. Cells were then harvested and centrifuged for 5 min at 100 *g* at 4 °C. The pelleted cells were resuspended in PBS with protease inhibitors cocktail (Sigma-Aldrich, Saint Louis, MI, USA) and lysed using slight sonication. SDS-PAGE was performed loading 10 μg of total protein extracts heated to 100 °C for 5 min in sample buffer (0.6 g/100 mL Tris, 2 g/100 mL SDS, 10% glycerol, 5% β-mercaptoethanol, pH 6.8). Proteins were electro-transferred to nitrocellulose membrane (cat. 1620115; Bio-Rad Laboratories, Hercules, CA, USA) using Trans-turbo transfer System (cat. 1704150; Bio-Rad Laboratories, Hercules, CA, USA). Filter Retardation Assay (FRA) was performed using a Bio-Dot SF Apparatus (Bio-Rad Laboratories, Hercules, CA, USA), as previously described (Sau *et al.* 2007). A 0.2-μm cellulose acetate membrane (Whatman 100404180) was treated in 20% methanol and washed in PBS. Then 3 μg (for BAG3-GFP) and 6 μg (for SOD1_G93A) of total protein were loaded and filtered by gentle vacuum. An equal amount of protein was loaded for each sample after correcting with a BCA assay. Then FRA membranes were washed in PBS and rinsed in 20% methanol. WB and FRA membranes were treated with a blocking solution of non-fat dried milk (5%) in TBS-Tween (20 mM Tris-HCl pH 7.5, 0.5 M NaCl, 0.05% Tween-20) for 1 h. WB and FRA membranes were then probed using the following antibodies: mouse monoclonal anti-GFP antibody (ab1218, Abcam, Cambridge, UK), mouse monoclonal anti-α-tubulin (T6199, Sigma-Aldrich, Saint Louis, MI, USA), homemade rabbit polyclonal anti-BAG3^14^, homemade rabbit polyclonal anti-HSPB8 (#23^52^), mouse monoclonal anti-HSPB8 (ab66063, Abcam, Cambridge, UK), rabbit polyclonal anti-Cu/Zn superoxide dismutase SOD1 (SOD-100, Enzo Life Sciences, Farmingdale, NY, USA), anti-ubiquitin (3936, Cell Signaling Technology, Danvers, MA, USA), rabbit polyclonal anti-BAG3 (10599-1-AP, Proteintech, Rosemont, IL, USA). Membranes were then washed 3 times for 10 min. Immunoreactivity was detected using the following secondary peroxidase conjugated antibodies: goat anti-rabbit and anti-mouse IgG-HRP (111-035-003, 115-035-003; Jackson ImmunoResearch Laboratories, Inc. Cambridge, UK) and enhanced chemiluminescent (ECL) detection reagent (Clarity ECL western blotting substrate, cat. 1705060; Bio-Rad Laboratories, Hercules, CA, USA). Images were acquired using a Chemidoc XRS System (Bio-Rad Laboratories, Hercules, CA, USA) and optical intensity was analyzed using Image Lab Software (Bio-Rad Laboratories, Hercules, CA, USA). Cropped blots are displayed in the manuscript, full uncropped images can be found as Supplementary Fig. S12.

### Flow Cytometric analysis of inclusions (FloIT)

HEK293T cells that were lentiviral transduced with HSPB8-V5 and were plated in 24-well plates at 75,000 cells/well. After 24 h, cells were transiently transfected using Lipofectamine3000/P3000 reagent, as previously described. After 48 h, medium was removed and cells were harvested in PBS with 10% FBS (Gibco, Thermo Fisher Scientific, Waltham, MA, USA) and centrifuged for 5 min at 100 *g* at 4 °C. Cells were resuspended in PBS with 10% FBS (Gibco, Thermo Fisher Scientific, Waltham, MA, USA) and an aliquot was analyzed by flow cytometry to determine the transfection efficiency in respect to untransfected control cells. Flow cytometry was performed using NovoCyte Flow Cytometer 3000 (ACEA Biosciences Inc., Agilent, Santa Clara, CA, USA) and results were analyzed by NovoExpress software 1.2.5 (ACEA Biosciences Inc., Agilent, Santa Clara, CA, USA). For transfection efficiency analysis, excitation wavelengths and emission collection windows were FITC (488 nm, 530/50 nm), Pacific Blue (405 nm, 445/45 nm). Subsequently, a solution of PBS containing 1% (v/v) Triton X-100, a cocktail of Protease inhibitors (Sigma-Aldrich, Saint Louis, MI, USA) and DAPI (0.02 µg/µl) was added to a final concentration of 0.5% (v/v) Triton X-100 and DAPI 0.01 µg/µl. After two minutes incubation at room temperature, the cell lysates were analyzed by flow cytometry. Three untransfected control samples without DAPI were analyzed to set gates on nuclei population. Voltage of 418 (FSC), 199 (SSC), 373/482 (FITC for cell transfection or inclusion analysis respectively), 501 (Pacific Blue) were used. Nuclei were counted based on the Pacific Blue positive population. Inclusions were identified for fluorescence and FSC compared to cells transfected with eGFPN1 vector as control. Following the equation set by Whiten *et al.* (2016) the number of inclusions was normalized to the number of counted nuclei and reported as inclusions/100 transfected cells. Nuclei population was analyzed based on FITC fluorescence and a percentage of nuclei enriched with GFP-positive particles was determined.

### Protein solubility predictions

To predict protein solubility, we used the CamSol browser (accessed on September 2^nd^ 2019; http://www-vendruscolo.ch.cam.ac.uk/camsolmethod.html) and Tango (accessed on September 3^rd^ 2019; http://tango.crg.es). As input for the CamSol method, we either inserted the full-length protein sequence of BAG3 (NP_004272.2) (Fig. S3) or the 20 amino acids surrounding the second IPV-motif (HQLPRGYISI**P**VIHEQNVTRP; Fig. 1d). In case of the latter, we also performed the solubility calculations for each of the respective mutants by replacing the Pro209 by either Ser, Leu or Gln. We used the CamSol Intrinsic method, as described in Sormanni *et al.*^32^.

For Tango, we inserted a protein sequence of 70 amino acids spanning the second IPV-motif (SQSPAASDCSSSSSSASLPSSGRSSLGSHQLPRGYISI**P**VIHEQNVTRPAAQPSFHQAQKTHYPAQQGEY)(Fig. 1e). The parameters were as following: no protection at the N-terminus or C-terminus of the peptide sequence, pH was selected as 7, temperature 298.15 K, ionic strength of 0.02 M, and a concentration of 1 M. We selected and plotted Beta-aggregation for both the wild type sequence as the three IPV-mutants (Ser/Leu/Gln).

### Co-immunoprecipitation

HEK293T stable cell lines for HSPB8-V5 were transiently transfected with different wild type or mutant BAG3-GFP constructs using PEI (23966-1, PolySciences Europe, Hirschberg an der Bergstrasse, Germany). Or alternatively, the reverse experiment was performed by transiently transfecting HeLa cells. After 48 h, cells were lysed with lysis buffer [20 mM Tris-HCl pH 7.4, 2.5 mM MgCl_2_, 100 mM KCl, 0.5% Nonidet P-40, Complete Protease inhibitor (Roche Applied Science, Indianapolis, IN, USA)] and incubated on ice for 30 min. Samples were centrifuged for 10 min at 20,000 *g* and equal amounts of supernatant (NP40-soluble fraction only) was loaded on GFP-Trap beads (gta-20, Chromotek, Martinsried, Germany). Beads were incubated with the protein lysate for 1 h at 4 °C and washed three times with wash buffer [20 mM Tris-HCl pH 7.4, 2.5 mM MgCl_2_, 100 mM KCl, Complete Protease inhibitor (Roche Applied Science, Indianapolis, IN, USA)]. Proteins were eluted from the beads with Sarkosyl elution buffer (140 mM NaCl, 50 mM Tris-HCl pH 8, 1 mM EDTA, 0.3% Sarkosyl, 10% glycerol) before being supplemented with NuPAGE LDS sample buffer (Life Technologies, Carlsbad, CA, USA) and loaded on 4-12% NuPAGE gels (Life Technologies, Carlsbad, CA, USA). Proteins were transferred to nitrocellulose membranes (Hybond-P; GE Healthcare, Wauwatosa, WI, USA) and decorated with antibodies against GFP (ab290, Abcam, Cambridge, UK), V5 (R96025, Invitrogen, Carlsbad, CA, USA), SQSTM1/p62 (5114, Cell Signaling, Danvers, MA, USA), Hsp70/Hsc70 (ab5439, Abcam, Cambridge, UK), or Tubulin (ab7291, Abcam, Cambridge, UK). Samples were detected using enhanced chemiluminescent ECL Plus (Pierce, Life Technologies, Carlsbad, CA, USA) and LAS4000 (GE Healthcare, Wauwatosa, WI, USA).

For the reverse experiment, co-immunoprecipitation of BAG3 after HSPB8 pull-down (Fig. S7) was performed as previously described in Minoia *et al.*^53^. In brief, HeLa cells were transfected using Lipofectamine 2000 reagent (Invitrogen, Carlsbad, CA, USA) with empty vector or BAG3-GFP constructs (wild type or mutants), according to manufacturer’s instructions. 24 h post-transfection cells were lysed in lysis buffer (150 mM NaCl, 0.5% NP40, 1.5 mM MgCl_2_, 20 mM Tris-HCl pH 7.4, 3% glycerol, 1 mM DTT, Complete Protease inhibitor (Roche Applied Science, Indianapolis, IN, USA)). The cell lysates were centrifuged and cleared with A/G beads (Santa Cruz Biotechnology, Inc., Santa Cruz, CA, USA) at 4 °C for 1 h. Rabbit TrueBlot beads (Tebu-bio) were incubated at 4 °C for 1 h with home-made rabbit HSPB8 antibody (Carra *et al.* 2005) or with rabbit serum (NRS), used as a control. Rabbit TrueBlot beads complexed with the specific antibodies were added to the precleared lysates. After incubation for 1 h at 4 °C, the immune complexes were centrifuged. Beads were washed four times with the lysis buffer; both co-immunoprecipitated proteins and input fractions were resolved on SDS-PAGE followed by western blot.

### Fluorescence microscopy and immunofluorescence

For quantification of protein aggregates, HEK293T stable cell line for HSPB8-V5, NSC-34 or C2C12 cells were plated in 24-well plates containing poly-D lysine (P-7280, Sigma-Aldrich, Saint Louis, MI, USA) coated coverslips and then transfected with wild type or mutant BAG3-GFP constructs as described above for WB and FRA experiments. For protein aggregation-prone behaviour evaluation, C2C12 were plated at 50,000 cells/ml, NSC-34 at 70,000 cell/ml in 24-well. Cells were then fixed using a 1:1 solution of 4% paraformaldehyde (PFA) and 4% sucrose in 0.2 N PB (0.06 M KH_2_PO_4_, 0.31 M Na_2_HPO_4_; pH 7.4) for 25 min at 37 °C. Nuclei were stained with Hoechst (1:2000 in PBS; 33342, Sigma-Aldrich, Saint Louis, MI, USA). Images were captured by Axiovert 200 microscope (Zeiss, Oberkochen, Germany) with a photometric CoolSnap CCD camera (Ropper Scientific, Trenton, NJ, USA). Images were processed using Metamorph software (Universal Imaging, Downingtown, PA). Six different fields were captured for each sample, of which each field of view contained an average of 95 cells. This summed up to a total of WT = 687 cells, Pro209Ser = 546 cells, Pro209Leu = 473 cells, Pro209Gln = 651 cells, Glu455Lys = 499 cells.

For colocalization studies of BAG3 and vimentin, the same HEK293T stable cell line for HSPB8-V5 were grown on poly-D-lysine (P7280-5×5MG, Sigma-Aldrich, Saint Louis, MI, USA) coated glass coverslips. Cells were transfected using PEI MAX (24765-1, PolySciences Europe, Hirschberg an der Bergstrasse, Germany) for wild type or mutant BAG3-GFP and fixed in ice-cold methanol (67-65-1, Sigma-Aldrich, Saint Louis, MI, USA) for 20 minutes. After blocking with 5% BSA (9048-46-8, Sigma-Aldrich, Saint Louis, MI, USA), cells were incubated with anti-GFP (Alexa Fluor 488 anti-GFP antibody; 338008, BioLegend, San Diego, CA, USA) and anti-vimentin (dilution 1:200; ab28028, Abcam, Cambridge, UK) for one hour at room temperature. After secondary antibody incubation, nuclei were stained with Hoechst33342 (H3570, Life Technologies, Carlsbad, CA, USA) and cells were mounted with DAKO fluorescent mounting medium (S3023, DAKO). Imaging was performed on a Zeiss LSM700 laser scanning confocal microscope using a 63×/1.4 NA objective. Image analysis was done in ImageJ/FIJI^54,55^.

For colocalization between BAG3 and SQSTM1/p62 or FLAG-HDAC6, HEK293T stable cell line for HSPB8-V5 were grown on polylysine-coated glass coverslip. Cells were then transfected with cDNAs encoding for wild type or mutant BAG3-GFP alone or, when indicated, FLAG-HDAC6^56^. After washing with cold PBS, cells were fixed with 3.7% formaldehyde in PBS for 9 minutes at room temperature, followed by permeabilization with cold acetone for 5 minutes at -20 °C. After blocking for 1 h at room temperature with BSA 3% and 0.1% Triton X-100, cells were incubated with anti-SQSTM1/p62 (sc-28359, Santa Cruz Biotechnology, Inc., Santa Cruz, CA, USA) or anti-FLAG (F3165, Sigma-Aldrich, Saint Louis, MI, USA) overnight at 4 °C. Cells were washed and incubated for 1 h at room temperature with a mouse secondary antibody (A21203, Life Technologies, Carlsbad, CA, USA); nuclei were stained with DAPI. Images were obtained using a Leica SP8 AOBS system (Leica Microsystems) and a 63x oil-immersion lens. Image analysis was done in ImageJ/FIJI^54,55^.

### Fluorescence recovery after photobleaching (FRAP) assay

HeLa cells were transfected with BAG3-GFP wild type or mutant constructs and with either P62-mCherry and mScarlet-HSP70 constructs and imaged 48 hours after transfection in a μ-slide 8-well (80826, Ibidi, Martinsried, Germany) in FluoroBrite DMEM medium (Life Technologies, Carlsbad, CA, USA) supplemented with 10% fetal bovine serum and 4mM L-glutamine at 37 °C and 5% CO_2_. FRAP measurements were performed on a Zeiss LSM700 laser scanning confocal microscope using a PlanApochromat 63×/1.4 NA objective.

Image sequences (512 ×512 pixels, 117 nm/pixel) were acquired at 1 frame per 3 sec (BAG3-GFP and mScarlet-HSP70 FRAP) or 1 frame per 2 sec (P62-mCherry FRAP) for the duration of the experiments, as indicated in the figures. Three to five pre-bleach sequences preceded photobleaching in a 70 ×70 pixel region at 100% of a 5 mW 488 nm laser (BAG3-GFP FRAP) or 100% of a 10 mW 555 nm (P62-mCherry and mScarlet-HSP70 FRAP) for 2 sec. FRAP sequences were recorded from 6 cells per genotype and intensities in the bleached region were measured with ImageJ^54,55^ and plotted over time. Imaging and photobleaching settings were kept identical for all wild type and mutant BAG3 cells within the three different FRAP experiments.

### Live-cell time-lapse imaging

HEK293T cells or HeLa cells were transfected with GFP-tagged BAG3 wild type or mutant constructs and imaged once per hour using an IncuCyte S3 instrument (Essen BioScience, UK). In case of co-localization experiments, cells were co-transfected with mCherry-tagged SQSTM1/p62 constructs (a kind gift from Prof. Sascha Martens (Max F. Perutz Laboratories, University of Vienna, Vienna Biocenter, Vienna, Austria)) or mScarlet-tagged Hsp70 constructs (we cloned Hsp70 into the pmScarlet_C1 plasmid, which was a kind gift from Dorus Gadella and available from Addgene (#85042)). Images were taken with a 20x objective at 37 °C and 5% CO_2_. GFP-tagged proteins were excited by a green laser for 300 ms and with a red laser for red-fluorescently tagged proteins for 400 ms. Images were exported and further analyzed in ImageJ^54,55^.

### Cycloheximide treatment

To determine protein turnover, a cycloheximide wash-out experiment was performed on HEK293T cells that stably overexpress HSPB8-V5. Cells were transiently transfected using PEI MAX (24765-1, PolySciences Europe, Hirschberg an der Bergstrasse, Germany) with wild type or mutant BAG3-GFP constructs. One day after transfection, cells were subjected to cycloheximide treatment (100 μg/ml) for either 6 h or 12 h. As a control, cells were subjected to dimethyl sulfoxide (DMSO) for 12 h. After the treatment, cells were collected in ice-cold PBS and proteins were extracted with RIPA buffer [1% Nonidet P-40, 150 mM NaCl, 0.1% SDS, 0.5% deoxycholic acid, 1 mM EDTA, 50 mM Tris-HCl pH 7.5, cOmplete Protease Inhibitor Cocktail (Roche Applied Science, Indianapolis, IN, USA), Phospho-STOP inhibitor mix (05 892 970 001, Roche Applied Science, Indianapolis, IN, USA)]. The soluble and insoluble fraction were separated by centrifugation at max speed for 10 minutes at 4 °C. Insoluble pellets were resuspended in sample buffer, while the soluble supernatant was subjected to a BCA assay (23225, Pierce BCA Protein Assay Kit) for protein quantification. Equal amounts of protein were loaded on 4–12% BisTris NuPAGE gels (Life Technologies, Carlsbad, CA, USA). Proteins were transferred to nitrocellulose membranes (Hybond-P; GE Healthcare, Wauwatosa, WI, USA). Ponceau S was used to assess equal loading of the insoluble fraction. Membranes were further decorated with antibodies against GFP (ab290, Abcam, Cambridge, UK), and β-Actin/ACTB (A5316, Sigma-Aldrich, Saint Louis, MI, USA). Samples were detected using enhanced chemiluminescent ECL Plus (Pierce, Life Technologies, Carlsbad, CA, USA) and LAS4000 (GE Healthcare, Wauwatosa, WI, USA). Protein densitometry was performed using ImageJ/FIJI^54,55^) and plotted over time relative to time-point zero.

### Autophagy assay

For western blot analysis of autophagic flux, HEK293T cells stably transduced with HSPB8-V5 were transiently transfected using PEI MAX (24765-1, PolySciences Europe, Hirschberg an der Bergstrasse, Germany) for wild type or mutant BAG3-GFP. After 24 h, cells were cultured in serum-deprived medium with bafilomycin A1 (10 nM) for two hours. Proteins were extracted with RIPA buffer [1% Nonidet P-40, 150 mM NaCl, 0.1% SDS, 0.5% deoxycholic acid, 1 mM EDTA, 50 mM Tris-HCl pH 7.5, cOmplete Protease Inhibitor Cocktail (Roche Applied Science, Indianapolis, IN, USA), Phospho-STOP inhibitor mix (05 892 970 001, Roche Applied Science, Indianapolis, IN, USA)], quantified using BCA (23225, Pierce BCA Protein Assay Kit). Equal amounts of protein were then loaded on 12% NuPAGE gels (Life Technologies, Carlsbad, CA, USA). Proteins were transferred to nitrocellulose membranes (Hybond-P; GE Healthcare, Wauwatosa, WI, USA) and decorated with antibodies against GFP (ab290, Abcam, Cambridge, UK), LC3B (L7543, Sigma-Aldrich, Saint Louis, MI, USA), and β-Actin/ACTB (A5316, Sigma-Aldrich, Saint Louis, MI, USA). Samples were detected using enhanced chemiluminescent ECL Plus (Pierce, Life Technologies, Carlsbad, CA, USA) and LAS4000 (GE Healthcare, Wauwatosa, WI, USA).

### Statistics

Statistical analyses have been performed using the statistical tests as stated in each figure legend. This comprised Student T tests or One-Way ANOVA with Bonferroni’s multiple comparisons tests. The statistical analysis was performed using PRISM software (GraphPad Software, La Jolla, CA, USA).

## Supplementary information

Supplementary information.
