# Supplementary material for: BAG3 Pro209 mutants associated with myopathy and neuropathy relocate chaperones of the CASA-complex to aggresomes
Source: Sci Rep. 2020 May 29;10:8755. doi: 10.1038/s41598-020-65664-z (PMC7260189; doi:10.1038/s41598-020-65664-z)

BAG3 Pro209 mutants associated with myopathy and neuropathy relocate chaperones of the CASA-complex to aggresomes

Elias Adriaenssens[1,5], Barbara Tedesco[2,5], Laura Mediani[3,5], Bob Asselbergh[4], Valeria Crippa[2], Francesco Antoniani[3], Serena Carra[3*], Angelo Poletti[2*], Vincent Timmerman[1*]

[1] Peripheral Neuropathy Research Group, Department of Biomedical Sciences, Institute Born Bunge, University of Antwerp, Antwerp, Belgium

[2] Dipartimento di Scienze Farmacologiche e Biomolecolari, Centro di Eccellenza sulle Malattie Neurodegenerative, Università degli Studi di Milano, Milano, Italy

[3] Department of Biomedical, Metabolic and Neural Sciences, University of Modena and Reggio Emilia, and Center for Neuroscience and Neurotechnology, Modena, Italy

[4] VIB-UAntwerp Center for Molecular Neurology, VIB and University of Antwerp, Antwerp, Belgium

[5] These authors contributed equally

[*] Correspondence: vincent.timmerman@uantwerpen.be (V.T.), angelo.poletti@unimi.it (A.P.), serena.carra@unimore.it (S.C.)

Supplementary Figures

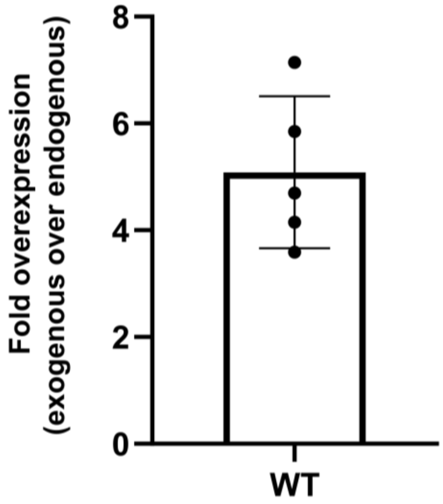

Fig. S1. Quantification of the relative fold overexpression of the exogenously GFP-tagged BAG3 over endogenously untagged BAG3. HEK293T-HSPB8-V5 cells were transiently transfected with GFP-tagged wild type BAG3 and protein lysates were analysed by western blot. With an anti-BAG3 antibody both the endogenous and exogenous BAG3 was visualized and the relative fold overexpression was determined with densitometric analysis of the western blot bands. (n=5)

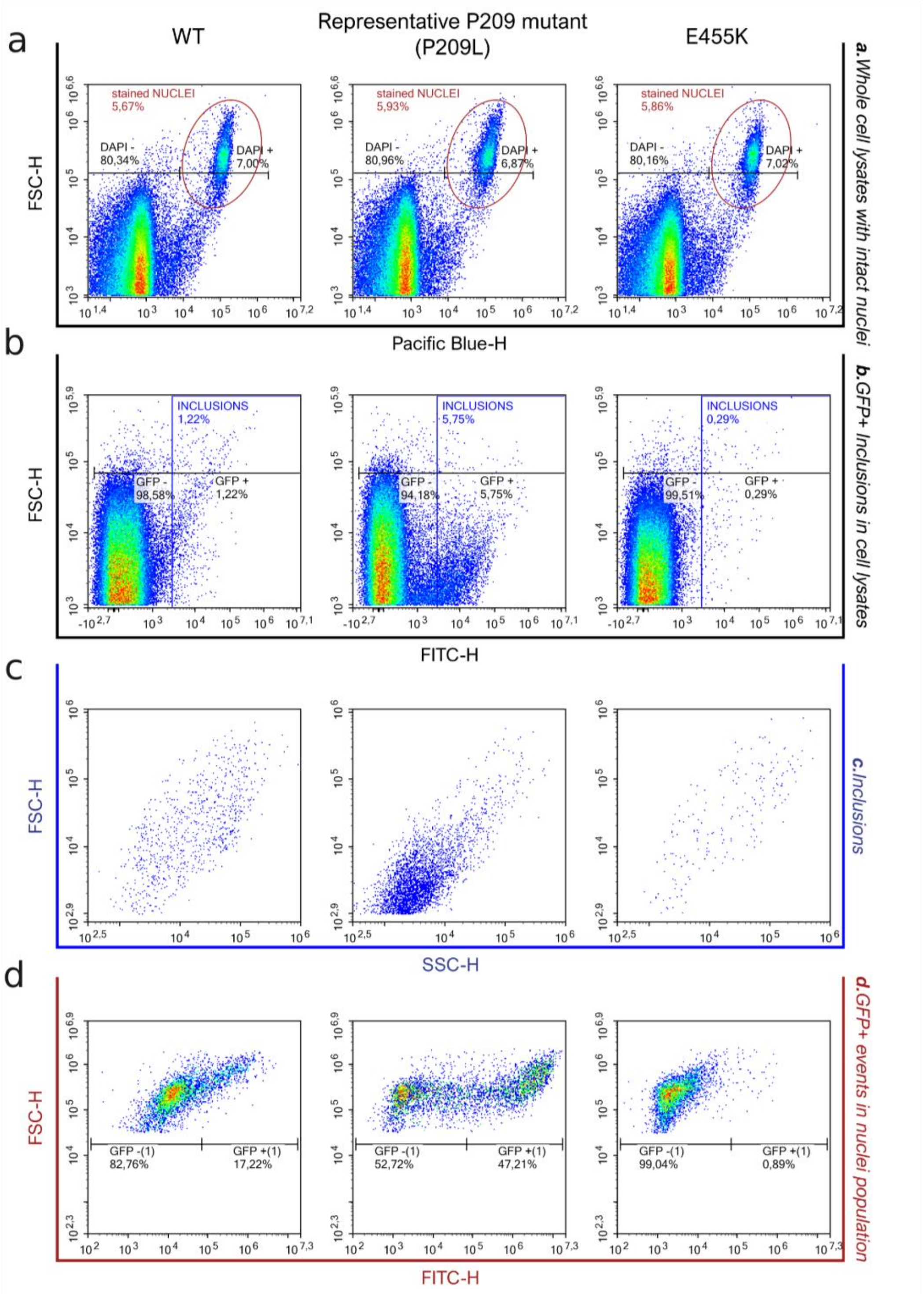

Fig. S2. Flow cytometric analysis of inclusions (FloIT) analysis for BAG3-GFP inclusions detection. HEK293T-HSPB8-V5 cells untransfected or transiently transfected with BAG3-GFP wild type or mutants were analysed by flow cytometry for transfection efficiency evaluation (γ = GFP+/cells). (**a**) After plasma membrane lysis, cellular lysates were read: nuclei populations were defined as Pacific Blue + populations after adding DAPI and excluded from cytoplasmic inclusion analysis. (**b**) The remaining particles were defined as GFP+ inclusions in respect to cells transfected with pEGFP-N1. (**c**) Distribution of GFP+ inclusions based on dimension and complexity. (**d**) Stained nuclei were analysed based on FITC fluorescence and dimension.

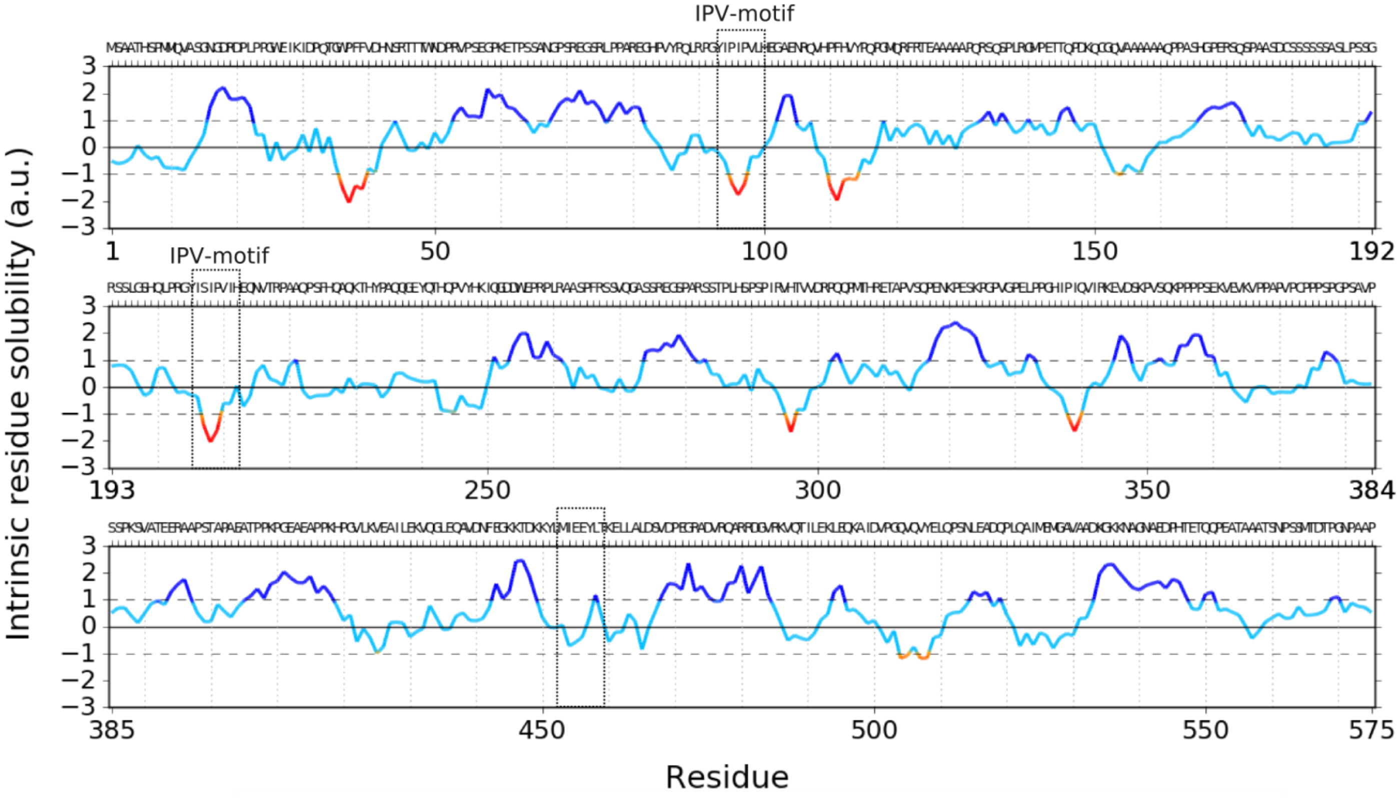

Fig. S3. Predicted residue solubility of full length BAG3. Bio-informatic solubility analysis of wild type BAG3 with CamSol software. As input we provided the first 575 amino acids of BAG3. The presented panels are the output as provided by the CamSol software and represent the predicted residue solubility. We highlighted the two IPV-motifs of BAG3 and the location of residue E455 in the BAG-domain.

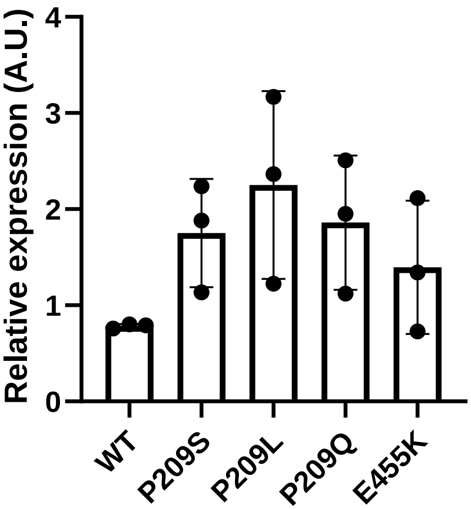

Fig. S4. Quantification of total amount of BAG3 wild type or mutants. HEK293T cells that stably overexpress HSPB8-V5 were transiently transfected with wild type or mutant BAG3-GFP constructs. Cells were collected in NP-40 containing buffer and the soluble fraction was separated from the NP-40 insoluble fraction. Both fractions were analysed by western blot and quantified using densitometric analysis. Samples were quantified relative to the wild type (WT). (n=3)

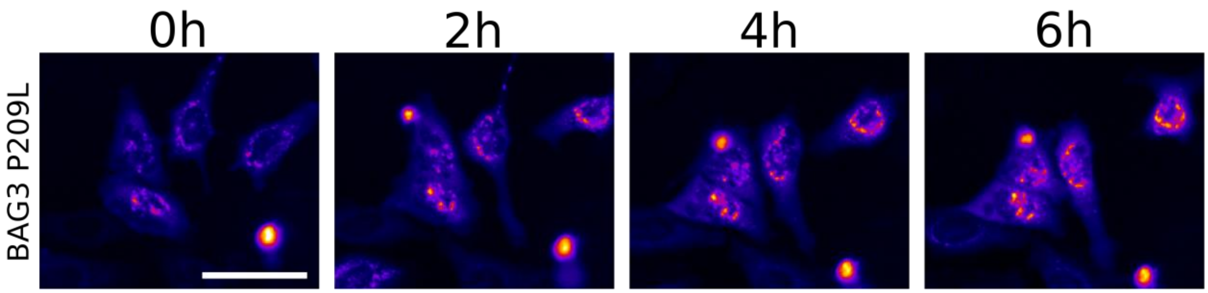

Fig. S5. Live-cell time-lapse imaging of GFP-tagged BAG3-Pro209Leu in HeLa cells. HeLa cells were transiently transfected with mutant BAG3-GFP constructs and imaged once per hour. Scale bar = 50 µm.

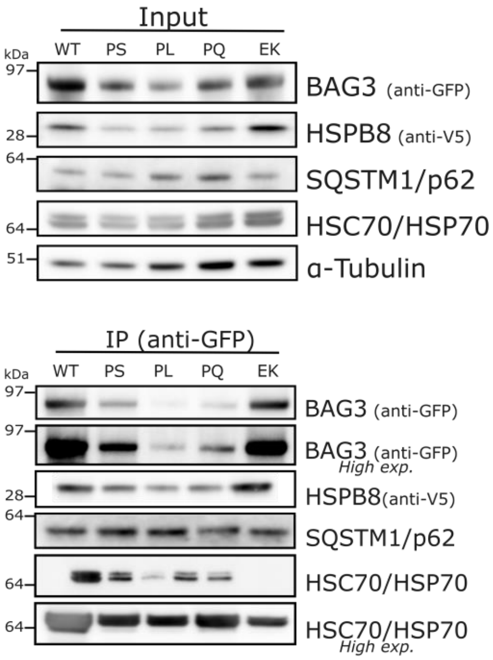

Fig. S6. Representative blots from the quantification shown in Figure 4a. HEK293T cells that stably overexpress HSPB8-V5 were transiently transfected with wild type or mutant BAG3-GFP constructs to assess the interaction between BAG3 and components of the CASA-complex. Co-immunoprecipitation of BAG3-GFP and the CASA-complex using the GFP-trap system. Both input and co-immunoprecipitating fraction is displayed. The wild type (WT) or mutants were abbreviated as followed: Pro209Ser (PS), Pro209Leu (PL), Pro209Gln (PQ), Glu455Lys (EK). The amount of interacting proteins was quantified and is displayed in Figure 4a.

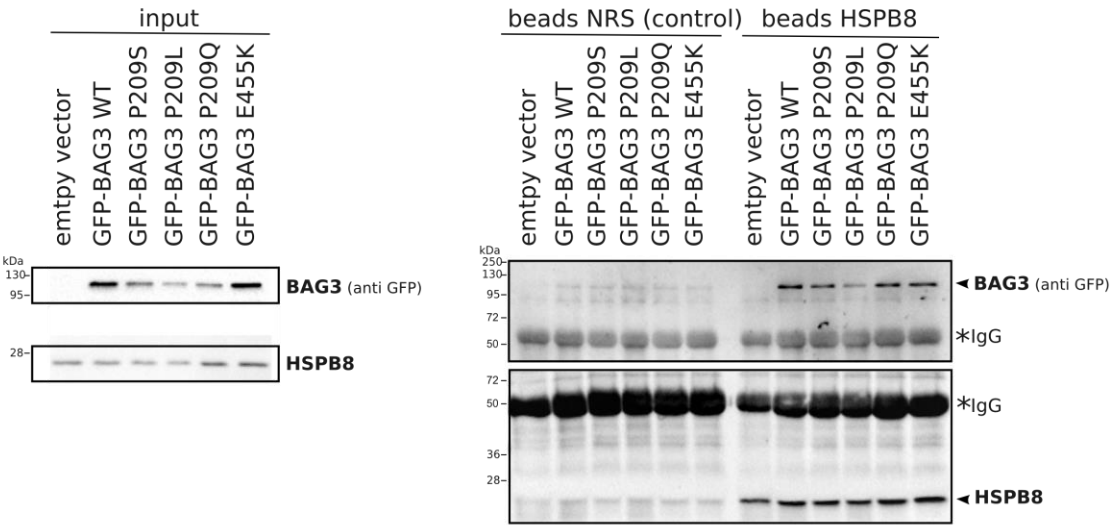

Fig. S7. Reverse co-immunoprecipitation of BAG3 after HSPB8 pull-down. HeLa cells were transiently transfected with wild type or mutant BAG3-GFP constructs. Beads coated with a specific anti-HSPB8 antibody or normal rabbit serum (NRS), used as negative control, were mixed with HeLa whole cell lysate. Both input and co-immunoprecipitation samples were loaded on SDS-PAGE. *IgG represents signal from the heavy chains. Arrowheads indicate the correct molecular weight for BAG3-GFP and HSPB8 respectively.

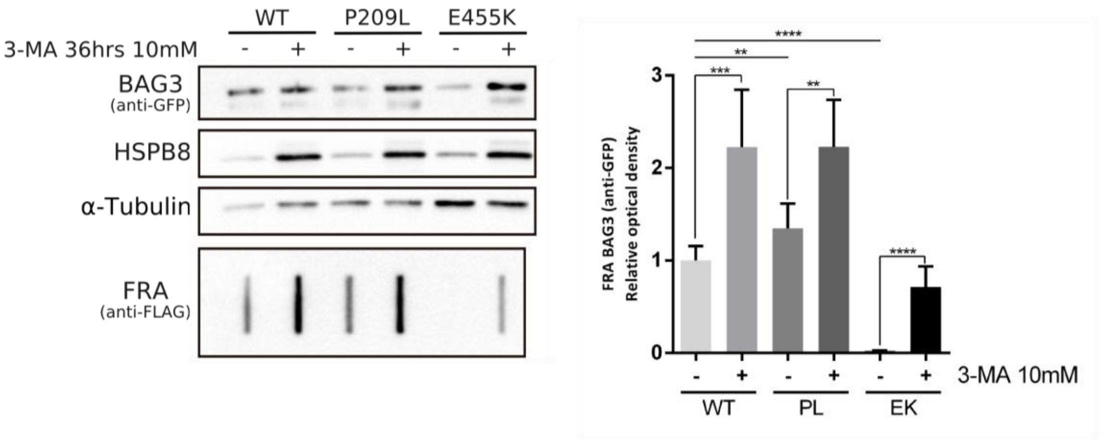

Fig. S8. Clearance assay of wild type and mutant BAG3 is prohibited by inhibition of autophagy. HEK293T cells that stably overexpress HSPB8-V5 were transiently transfected with mutant BAG3-GFP constructs. Cells were left untreated or treated with 3-MA for 36 hours after which protein lysates were collected and analysed by western blot and filter retardation assay (FRA). The FRA analysis is displayed for the NP-40 insoluble fraction. Relative optical densities are reported in the graph as means ± SD of normalized values. Student T tests were used for statistical analysis to compare the treated with untreated condition for each respective BAG3 variant (n=3).

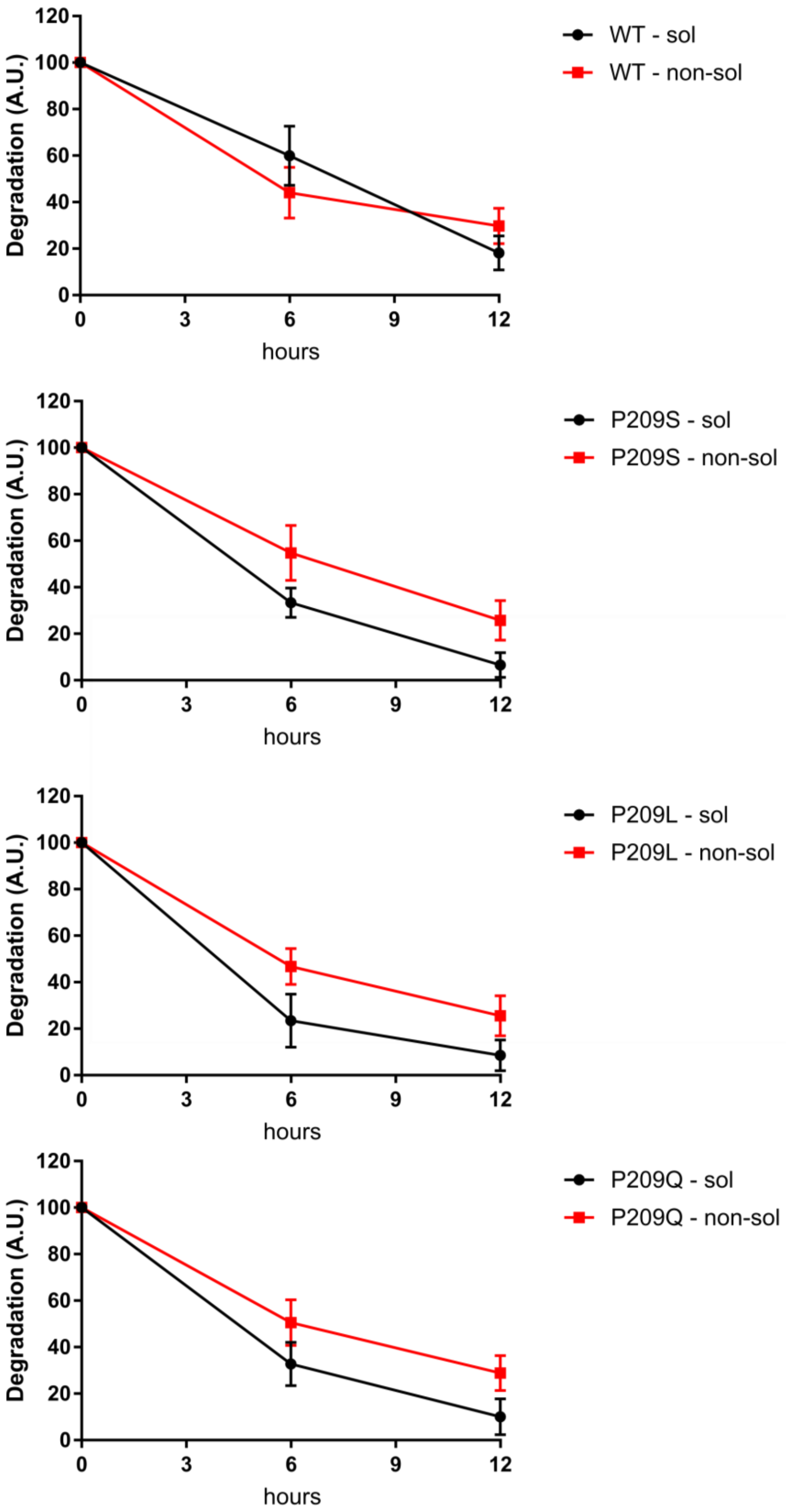

Fig. S9. Protein degradation rate soluble versus nonsoluble. The same protein turnover results from Figure 3b are presented here as soluble versus nonsoluble for each of the respective genotypes. The wild type (WT) or mutants were abbreviated as followed: Pro209Ser (PS), Pro209Leu (PL), Pro209Gln (PQ). Data are presented as mean±SD. (n=3)

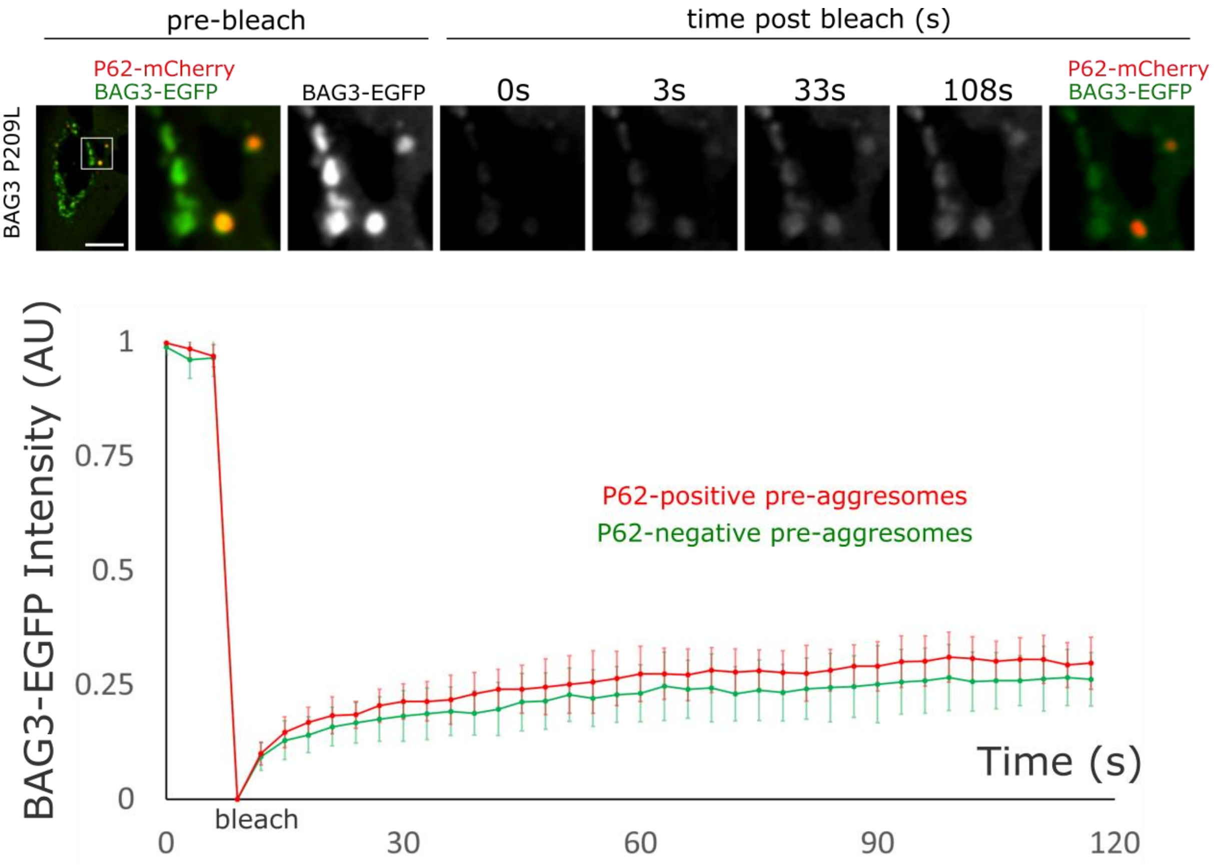

Fig. S10. The mobility of BAG3_Pro209 mutants is indifferent in SQSTM1/p62-positive versus SQSTM1/p62-negative pre-aggresome bodies. Fluorescence recovery after photobleaching (FRAP) analysis was performed on HeLa cells that were transiently transfected with BAG3-GFP and SQSTM1/p62-mCherry constructs. Quantification of the fluorescence intensity was plotted over time for mutant BAG3_Pro209Leu cells. Graph bar shows the means (± SD) over time (n=4). Scale bar = 10 µm

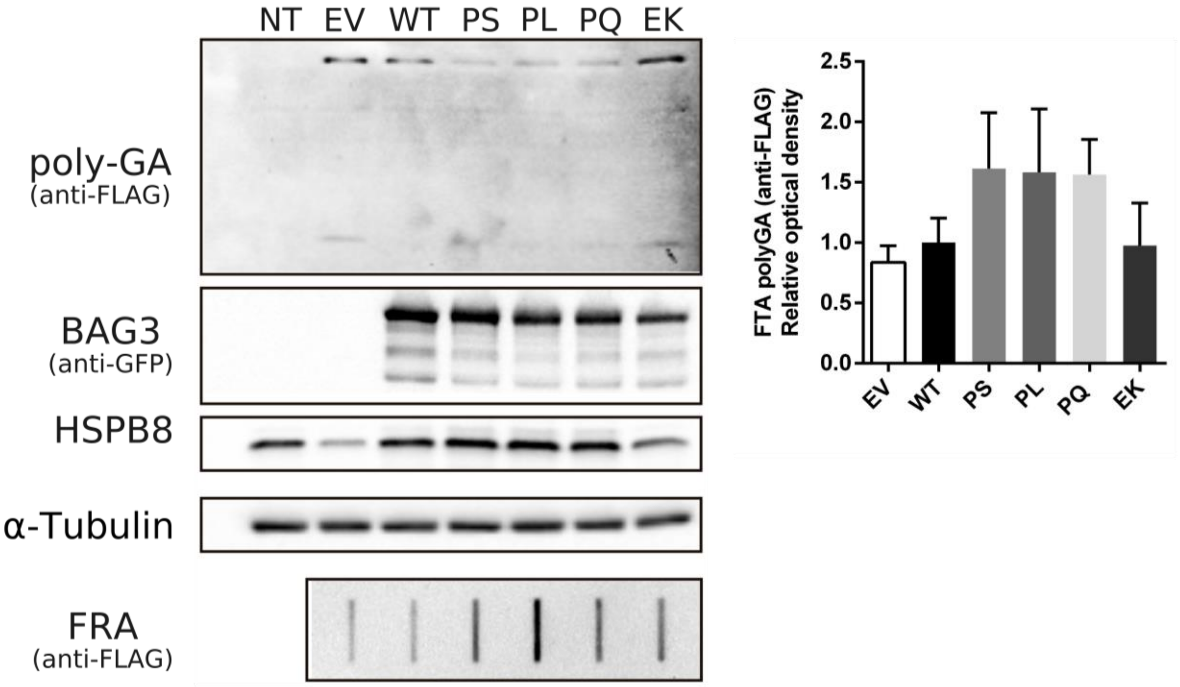

Fig. S11. Clearance assay of neurotoxic polyGA dipeptide repeat proteins by wild type and mutant BAG3 complexes. HEK293T cells that stably overexpress HSPB8-V5 were transiently transfected with mutant BAG3-GFP constructs and FLAG-tagged polyGA. Protein lysates were collected and analysed for polyGA aggregation using a filter retardation assay (FRA). Abbreviations: non-transfected (NT), empty vector (EV), wild type (WT), Pro209Ser (PS), Pro209Leu (PL), Pro209Gln (P209Q), Glu455Lys (E455K), filter retardation assay (FRA). (n=6)

Fig. S12. Unprocessed images of all blots.
Uncropped full blots are displayed of figures 1f, 1g, 2a, 2c, 4a and S6, 5a, 6a, 6c, 6d, 7a, 7c, S7, S8, S11.

<u>Figure 1f</u>

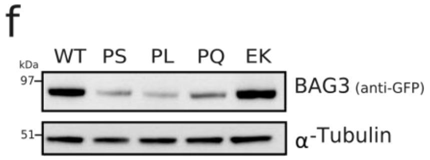

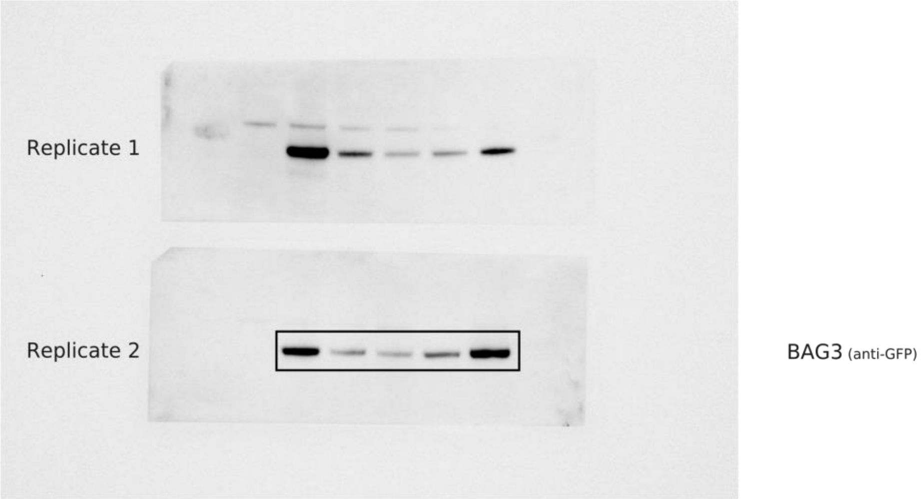

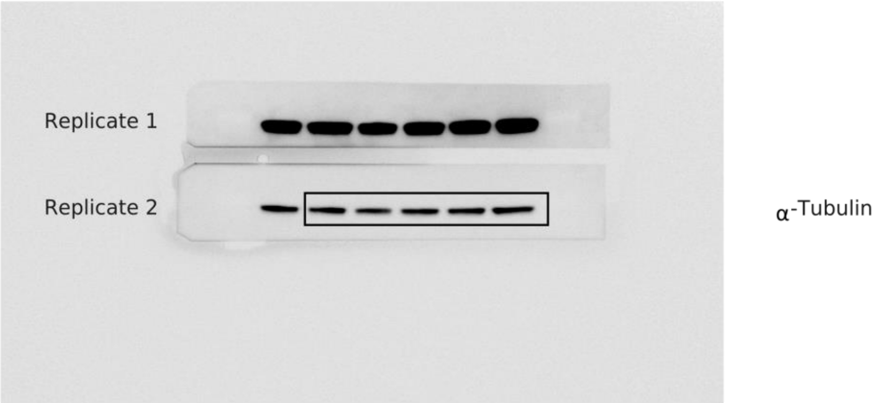

Fig. S12. Unprocessed images of all blots (continued).

Figure 1g

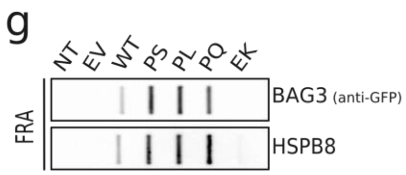

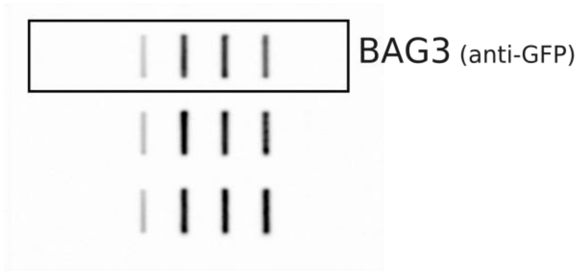

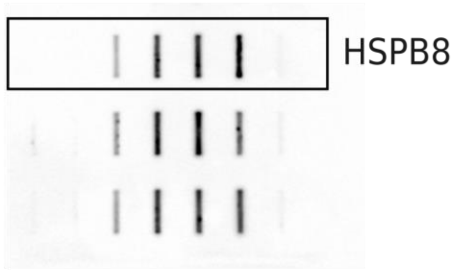

Fig. S12. Unprocessed images of all blots (continued).

Figure 2a

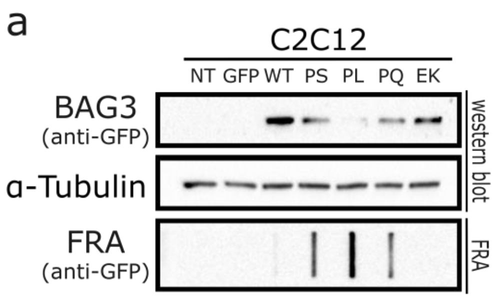

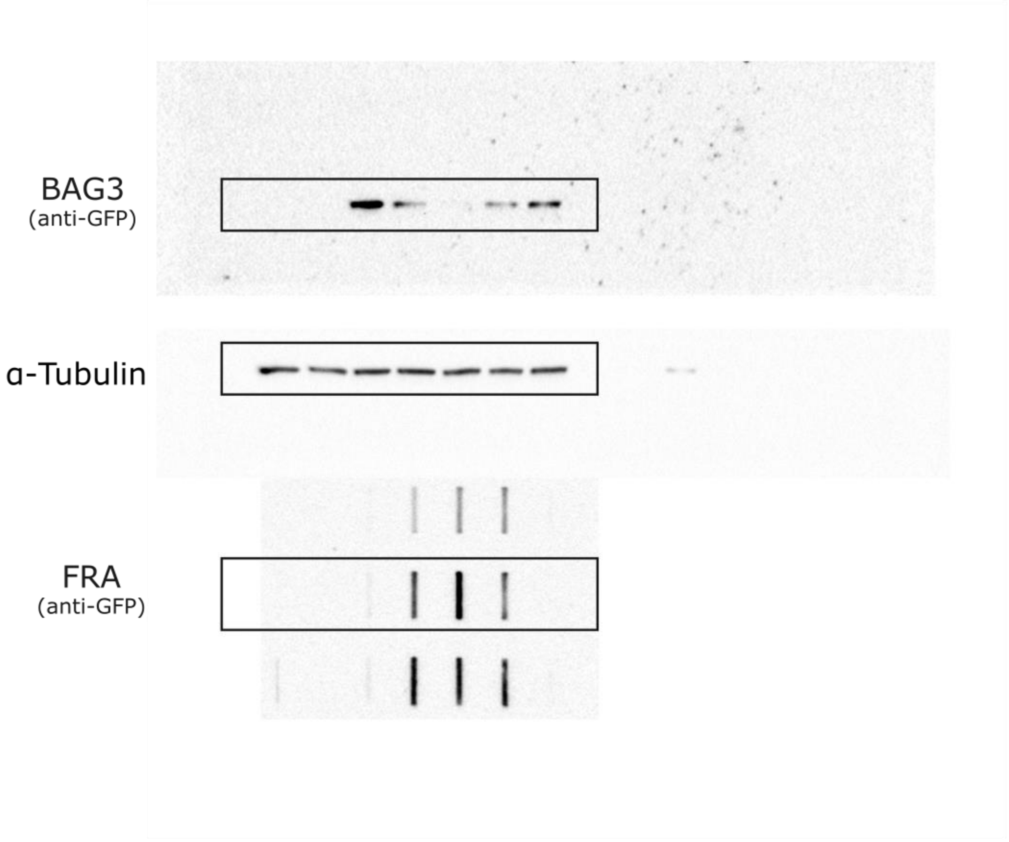

Fig. S12. Unprocessed images of all blots (continued).

<u>Figure 2c</u>

c

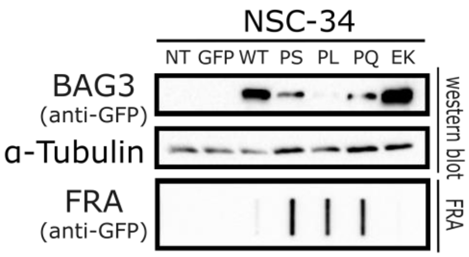

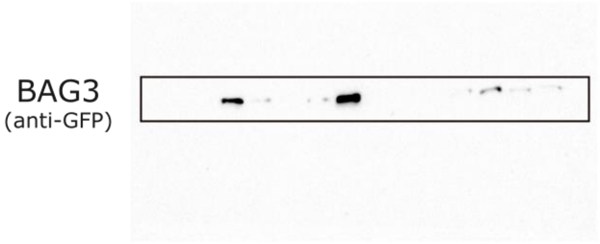

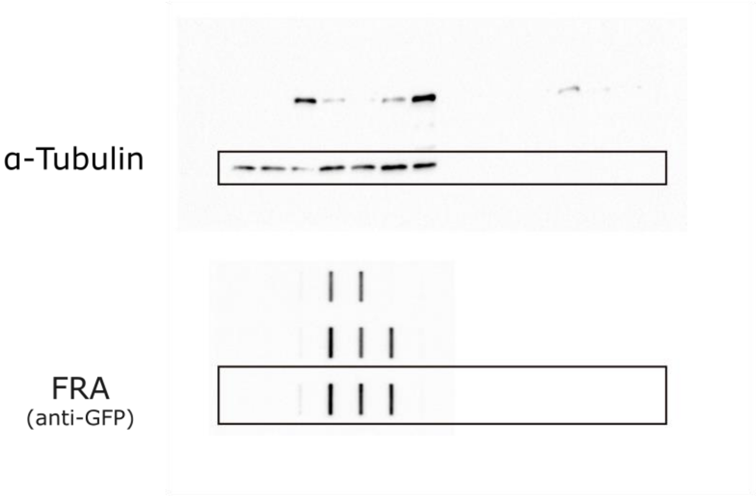

Fig. S12. Unprocessed images of all blots (continued).

Figures corresponding to the quantification in Figure 4a and which are displayed in Figure S6

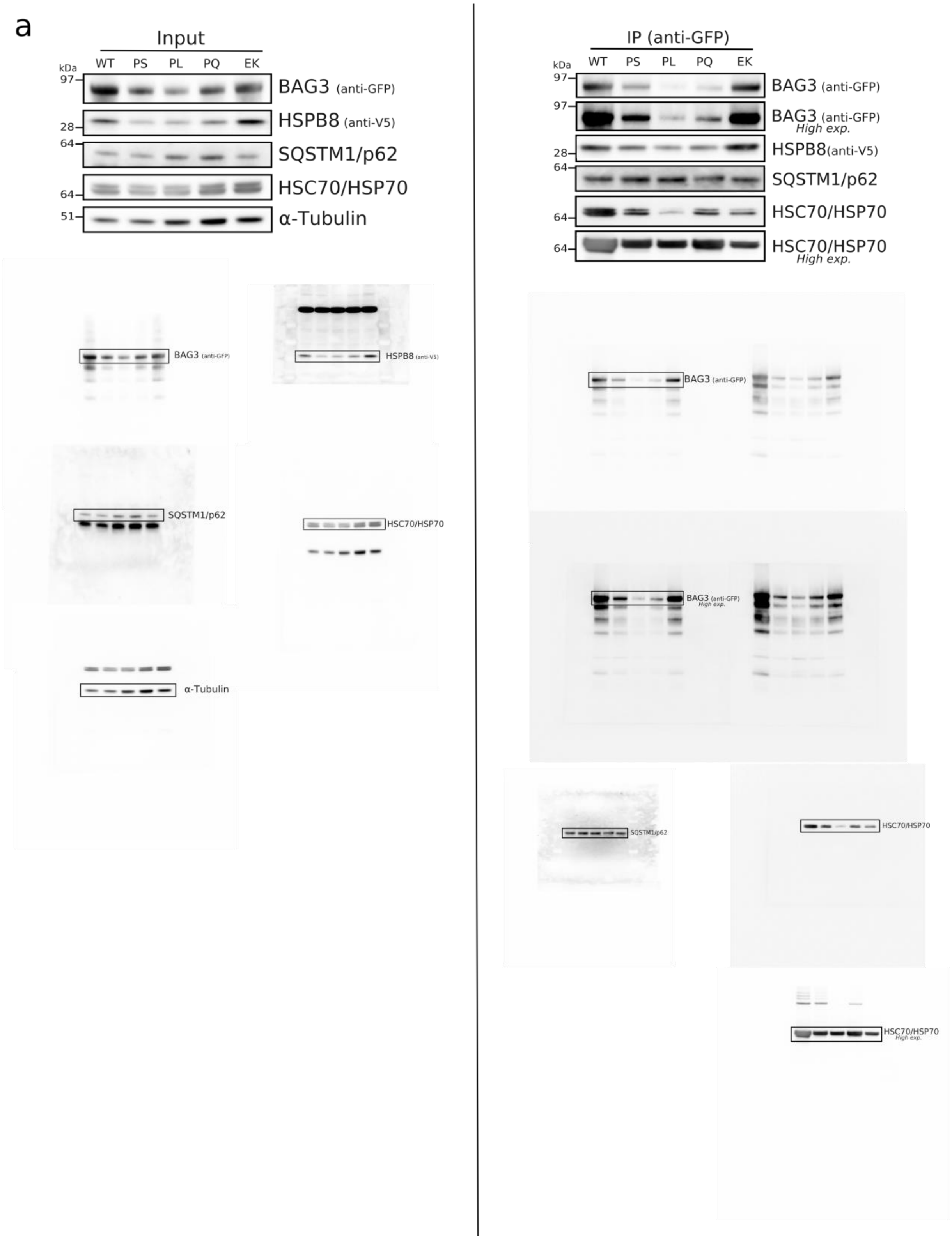

Fig. S12. Unprocessed images of all blots (continued).

Figure 5a

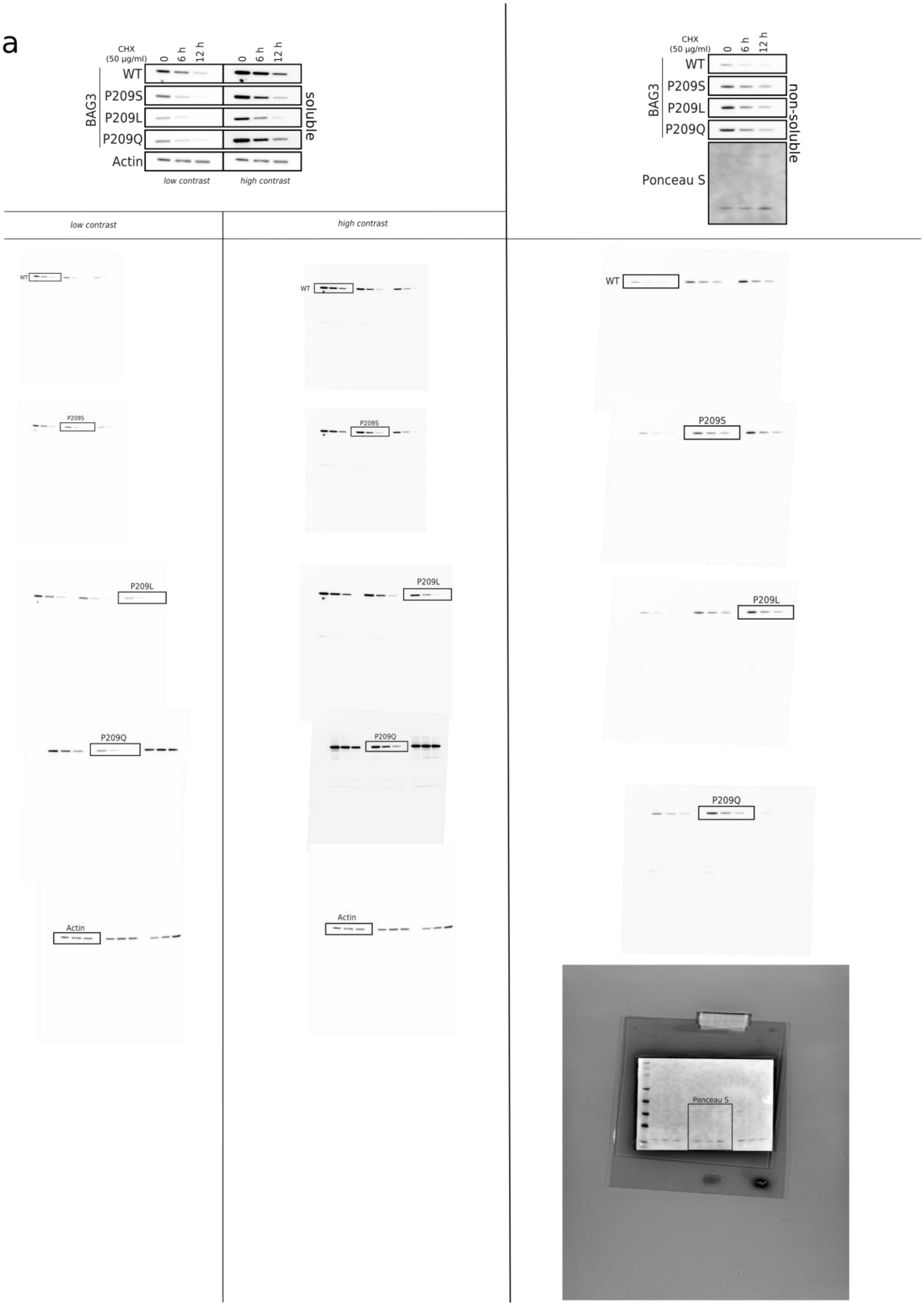

Fig. S12. Unprocessed images of all blots (continued).

Figure 6a

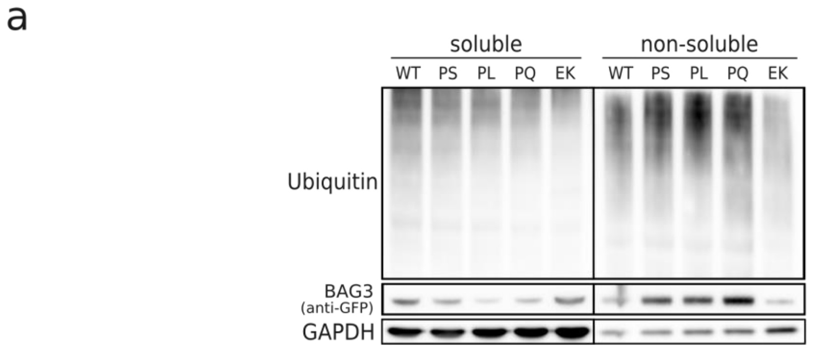

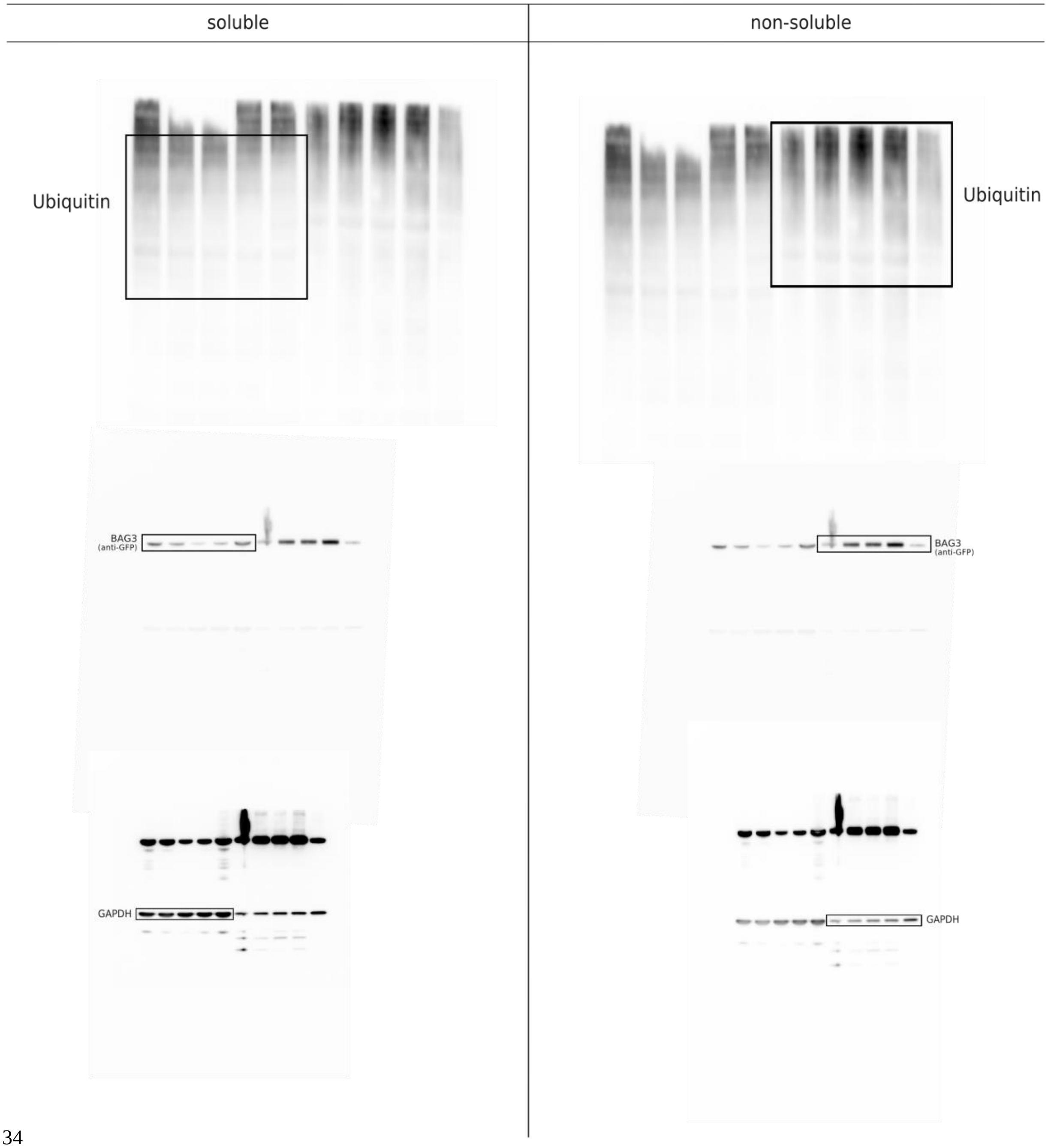

Fig. S12. Unprocessed images of all blots (continued).

<u>Figure 6c</u>

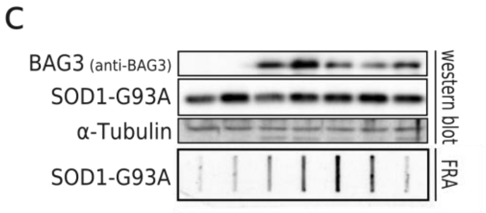

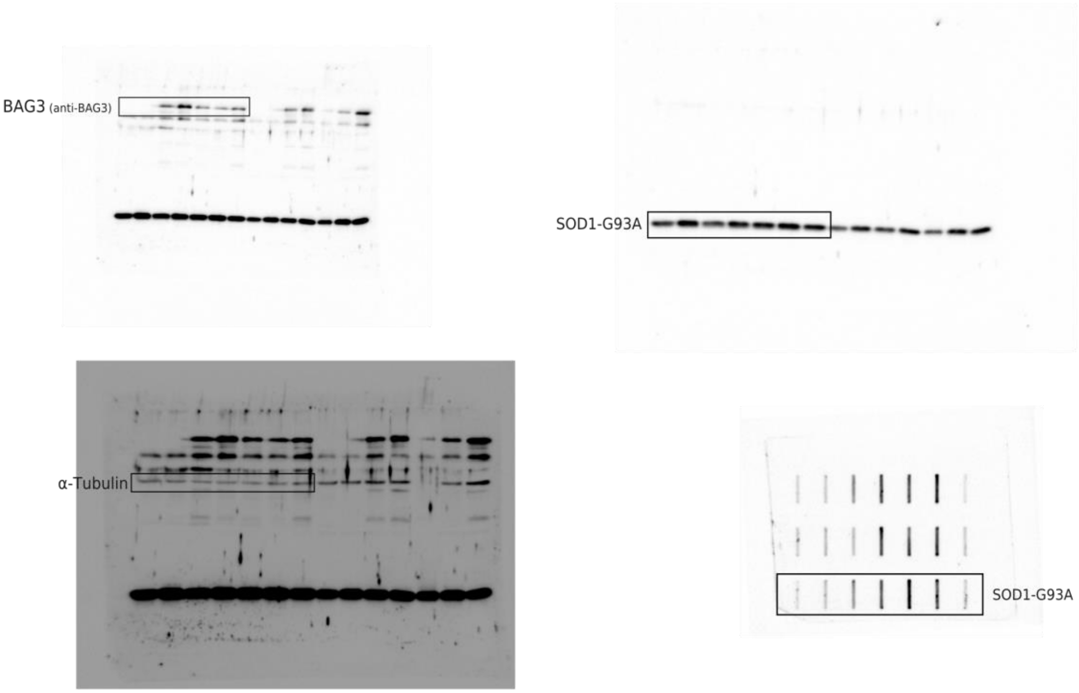

Fig. S12. Unprocessed images of all blots (continued).

Figure 6d

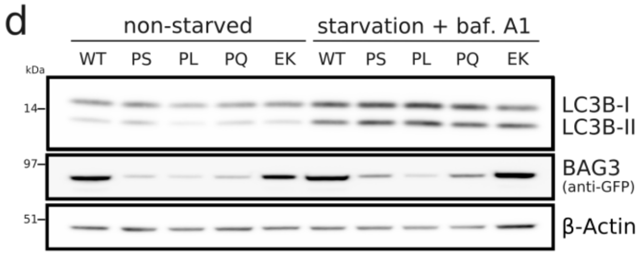

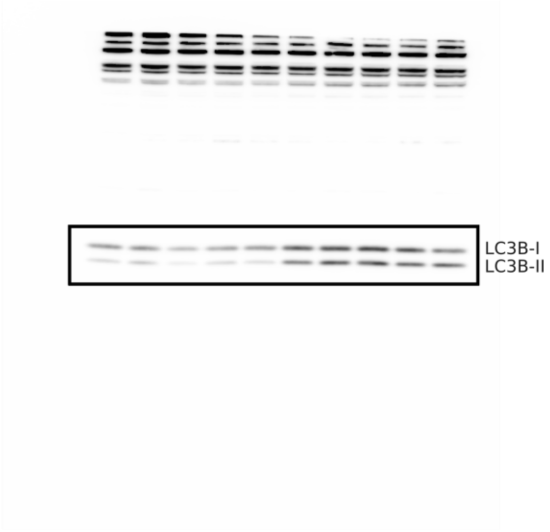

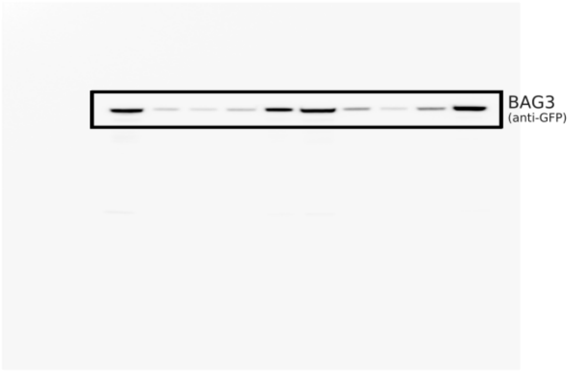

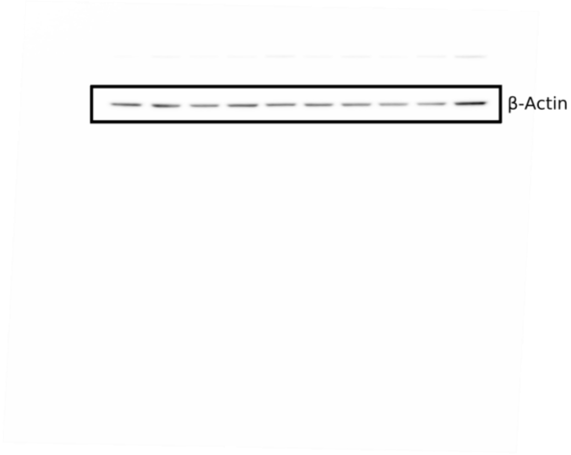

Fig. S12. Unprocessed images of all blots (continued).

Figure 7a

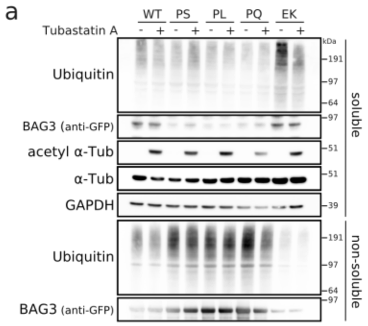

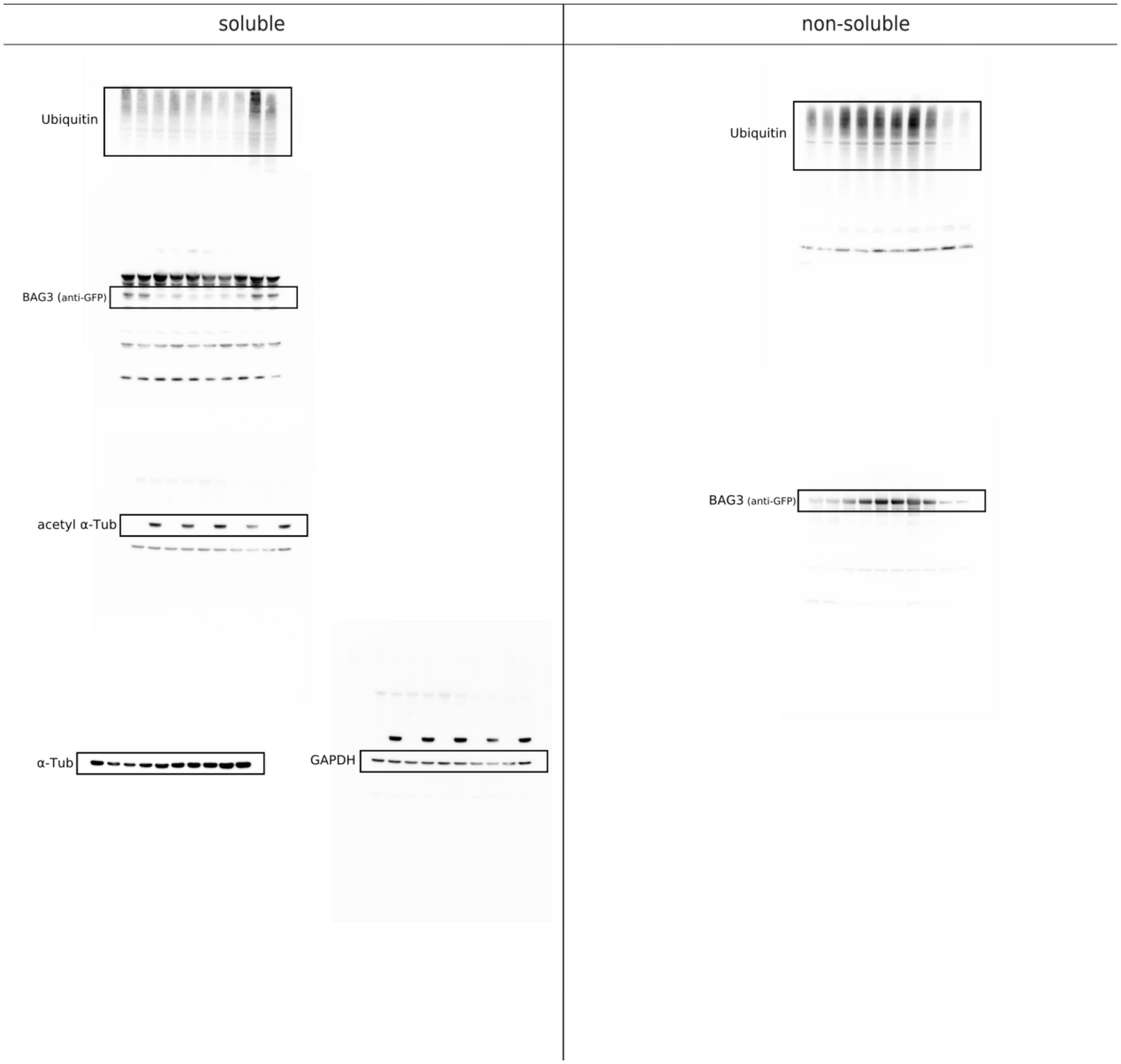

Fig. S12. Unprocessed images of all blots (continued).

Figure 7c

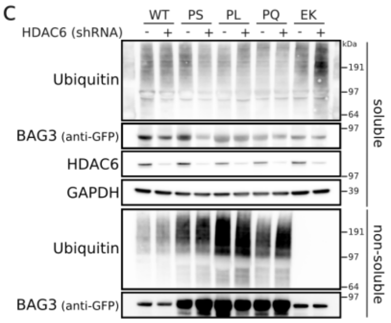

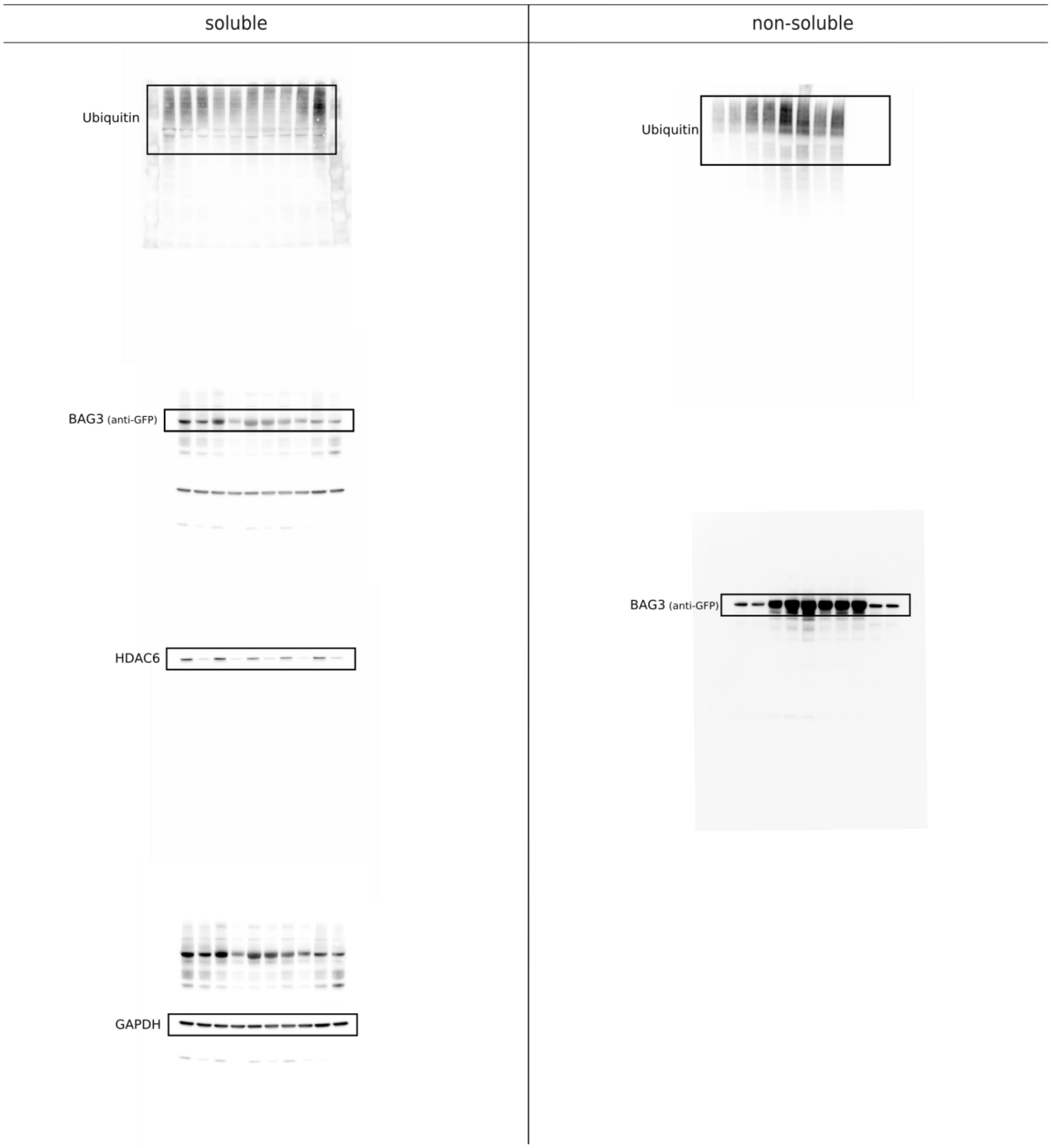

Fig. S12. Unprocessed images of all blots (continued).

Figure S7

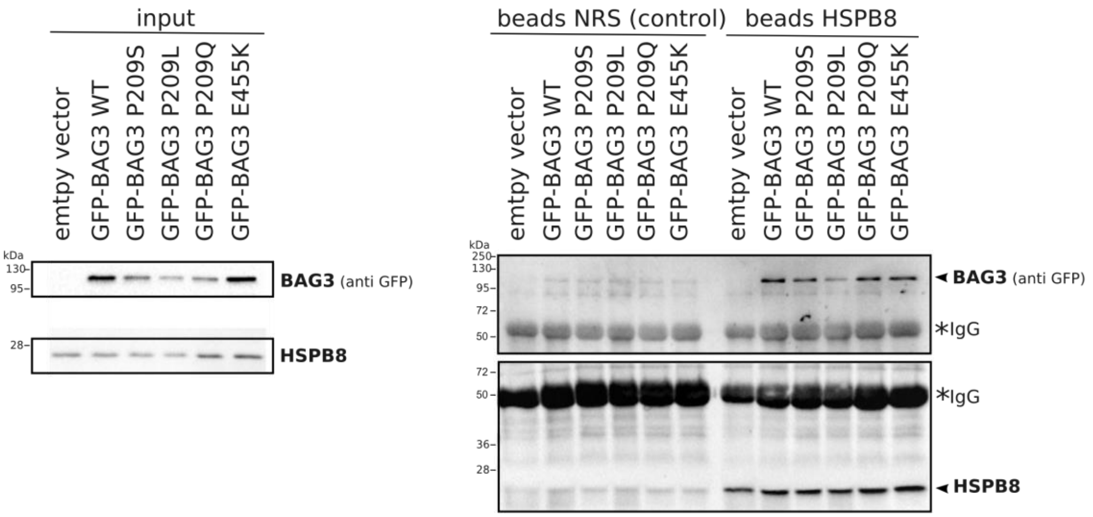

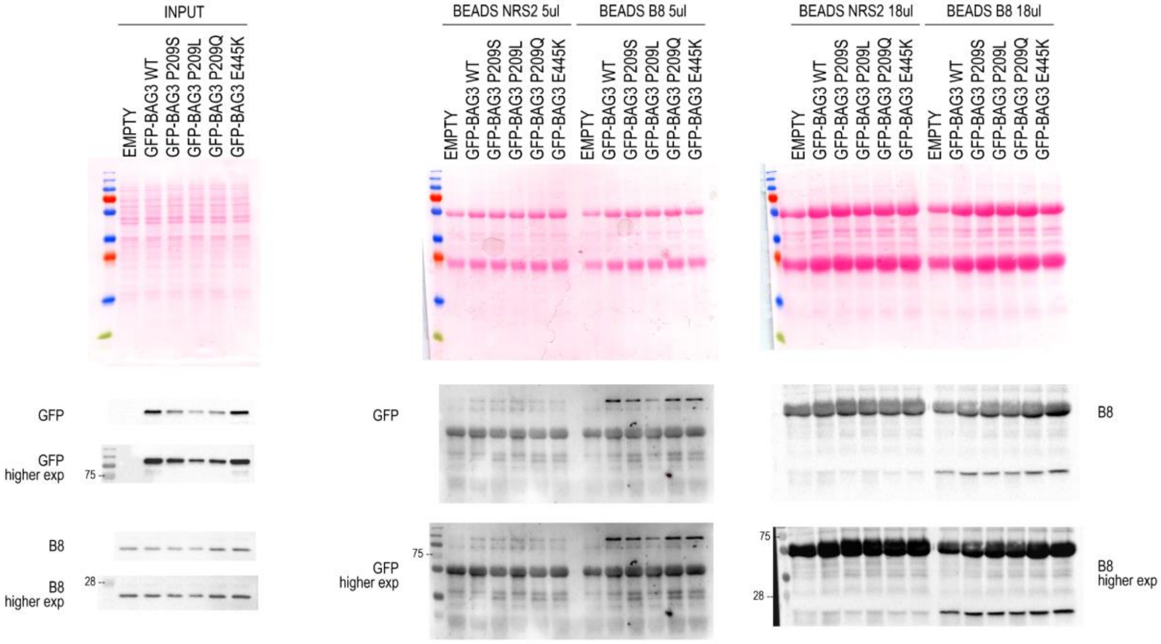

Fig. S12. Unprocessed images of all blots (continued).

Figure S8

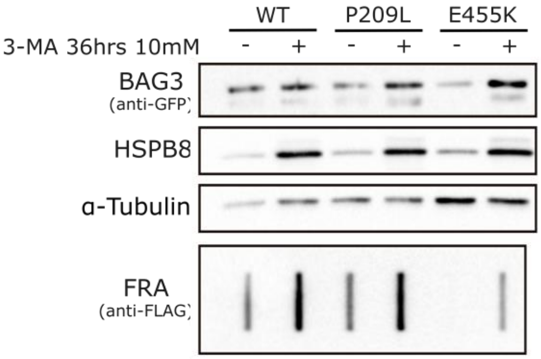

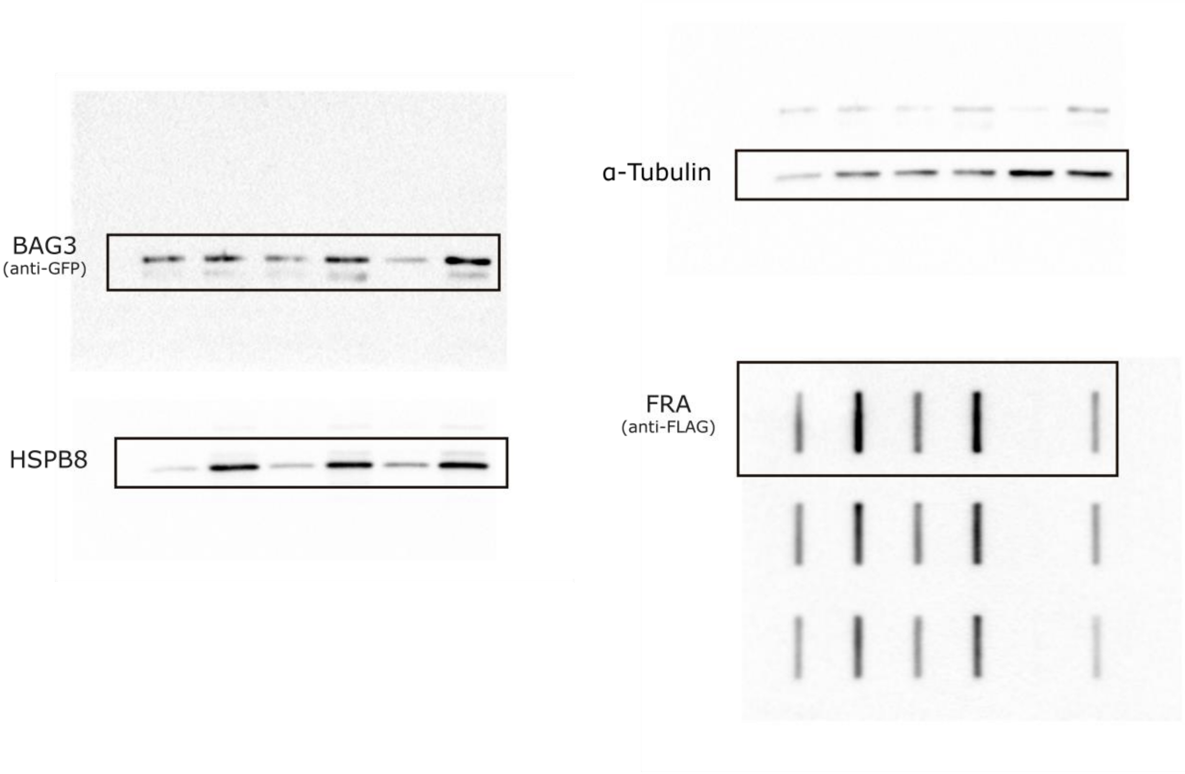

Fig. S12. Unprocessed images of all blots (continued).

Figure S11

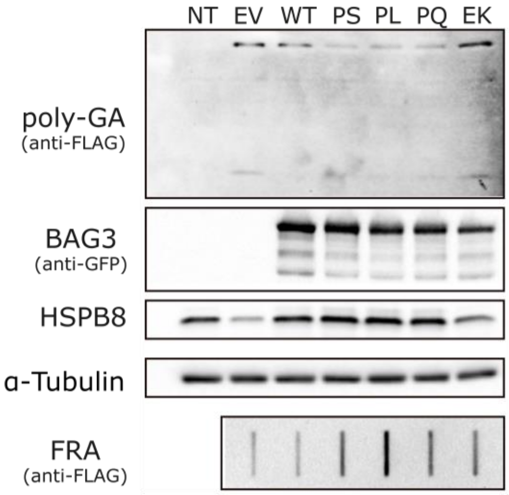

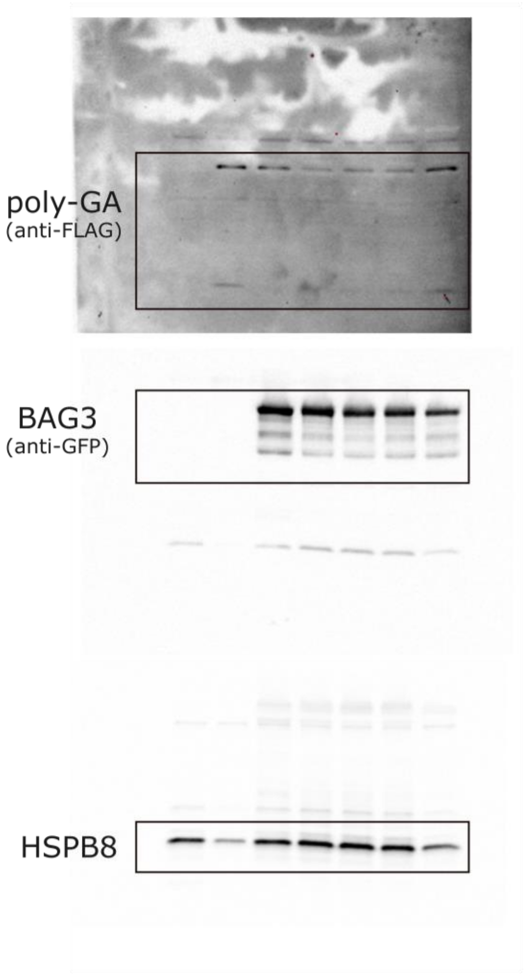

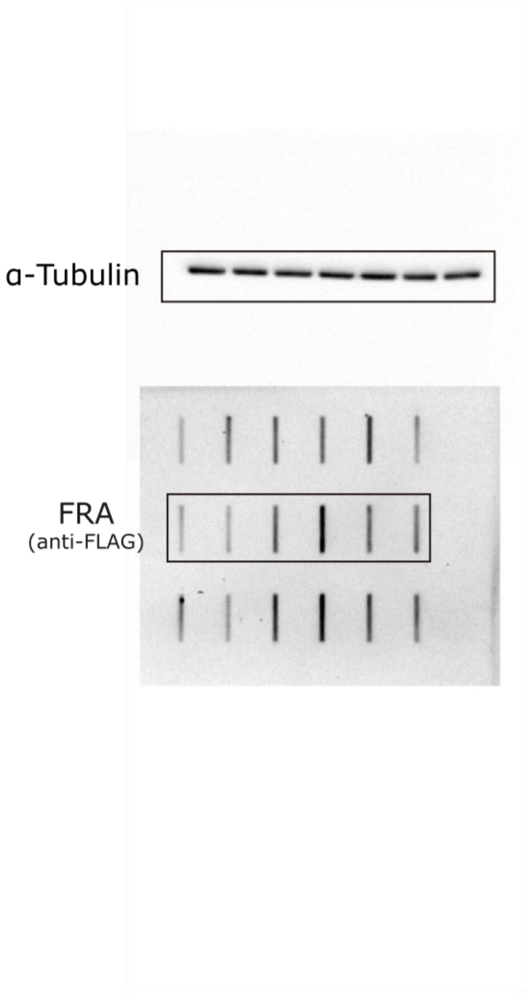

Supplement: Supplementary file 1 — Supplementary information. [file 41598_2020_65664_MOESM1_ESM.pdf]
